# Next-Generation Minimally Invasive Anti-Aging Therapy: Incorporation of Resveratrol-Nicotinamide Cerosomes into PLGA Microneedles for Enhanced Skin Permeation

**DOI:** 10.3390/pharmaceutics18030326

**Published:** 2026-03-04

**Authors:** Sammar Fathy Elhabal, Mai S. Shoela, Fatma E. Hassan, Suzan Awad AbdelGhany Morsy, Amal M. Elsharkawy, Amany Ali Khalil Nawar, Mona Mohamed Ahmed, Shady Allam, Marwa A. Fouad, Amal Anwar Taha, Ahmed Mohsen Faheem, Hanan Mohamed Abd Elmoneim, Ahmed Mohsen Elsaid Hamdan

**Affiliations:** 1 Department of Pharmaceutics and Industrial Pharmacy, Faculty of Pharmacy, Modern University for Technology and Information (MTI), Mokattam, Cairo 11571, Egypt; 2 Department of Clinical Pharmacology, Faculty of Medicine, Alexandria University, Alexandria 21526, Egypt; 3 Department of Physiology, General Medicine Practice Program, Batterjee Medical College, Jeddah 21442, Saudi Arabia; 4 Medical Physiology Department, Faculty of Medicine, Cairo University, Kasr Alainy, Giza 11562, Egypt; 5 Pathological Sciences Department, MBBS Program, Fakeeh College for Medical Sciences, Jeddah 21461, Saudi Arabia; 6 Department of Biophysiology, Ibn Sina National College for Medical Studies, Jeddah 22421, Saudi Arabia; 7 Department of Pharmacology and Toxicology, Faculty of Pharmacy, Menoufia University, Menoufia 32811, Egypt; 8 Department of Pharmacology and Toxicology, Faculty of Pharmacy, Menoufia National University, Cairo-Alexandria Agricultural Road, Menoufia 32511, Egypt; 9 Department of Pharmaceutics and Pharmaceutical Technology, Faculty of Pharmacy, Deraya University, Minia 61768, Egypt; 10 Department of Pharmaceutics, College of Pharmaceutical Sciences and Drug Manufacturing, Misr University for Science and Technology (MUST), 6th of October City 12566, Egypt; 11 Department of Medical Biochemistry and Molecular Biology, Faculty of Medicine, Mansoura University, Mansoura 35516, Egypt; 12 Department of Pathology, Faculty of Medicine, Umm Al-Qura University, Makkah 24381, Saudi Arabia; 13 Department of Pathology, Faculty of Medicine, Minia University, Minia 61519, Egypt; 14 Department of Pharmacology and Toxicology, Faculty of Pharmacy, University of Tabuk, Tabuk 71491, Saudi Arabia; 15 Prince Fahad bin Sultan Chair for Biomedical Research (PFSCBR), Tabuk 74191, Saudi Arabia

**Keywords:** anti-wrinkle therapy, cerosomes, nicotinamide, PLGA, resveratrol, skin aging, microneedle-based systems, skin delivery

## Abstract

**Background/Objectives**: Skin aging and wrinkle formation are primarily driven by ultraviolet (UV)-induced oxidative stress and inflammation. Resveratrol (RSV) and nicotinamide (NCT) possess potent anti-aging properties but suffer from poor skin penetration. This study aimed to develop an advanced transdermal delivery system incorporating RSV/NCT-loaded cerosomes within poly(lactic-co-glycolic acid) (PLGA) microneedles to enhance skin permeation and anti-aging performance. **Methods**: RSV/NCT-loaded cerosomes were formulated using thin-film hydration of phosphatidylcholine, ceramides (III, IIIB, and VI), and poloxamer surfactants, subsequently optimized via a D-optimal mixture design. PLGA microneedles with optimized cerosomes were tested for their mechanical strength, penetration, drug loading, and release. Ex vivo permeation and in vivo evaluations were performed using a UVA-induced skin wrinkling model. **Results**: Optimized cerosomes exhibited high entrapment efficiency for RSV and NCT (91 ± 0.56% and 85 ± 0.56%, respectively), nanoscale size (195 ± 0.78 nm), low polydispersity (0.23 ± 0.01), and a negative zeta potential (−22 ± 0.45 mV). PLGA microneedles exhibited sufficient mechanical integrity and effective penetrability through Parafilm^®^ layers. Microneedle-loaded cerosomes enabled sustained drug release (approximately 65–70% over 48 h) and enhanced ex vivo permeation, approximately for NCT and RSV (1450 μg/cm^2^ and 1000 μg/cm^2^, respectively). In vivo investigations revealed improved skin appearance, restoration of epidermal thickness and collagen architecture, reduced levels of inflammatory cytokines (TNF-α, IL-1β, IL-6, NLRP3), reduced oxidative stress biomarkers (GSH, GPx, MDA, SOD), and genetic upregulation of VEGF, TGF-β1, and β-Catenin. **Conclusions**: The RSV/NCT cerosome-encapsulated PLGA microneedle system offers a promising, minimally invasive approach with superior transdermal delivery, sustained efficacy, and significant anti-aging benefits.

## 1. Introduction

The skin is the largest organ in the human body and acts as the first line of defense against environmental stressors, such as pollution and the sun’s ultraviolet (UV) radiation [1,2]. Continuous exposure to these stressors results in premature skin aging and the development of wrinkles and uneven skin tone. Some skin conditions result from environmental stressors, leading to skin inflammation [3,4]. Dermal wrinkles form as the physiological activities and structures of the skin change. Aging of the skin is caused by intrinsic and external factors. Intrinsic aging results in fine wrinkles, skin dryness, and reduced skin elasticity. On the other hand, external aging of the skin is caused by air pollution and exposure to the sun, leading to the formation of more wrinkles and inflamed skin [5]. Ultraviolet light from the sun is the main cause of skin damage and the formation of free radicals, which results in increased reactive oxygen species (ROS) which alters lipids, proteins, and DNA [6]. Additionally, as people age, the skin’s ability to damage and repair itself declines. Some skin aging changes, such as decreased androgen production, reduced skin elasticity, increased dryness, and more wrinkles, are the result of reduced sebum production [6,7]. One study identified that inflammatory skin conditions and microbial pathogens due to skin barrier damage may stimulate systemic inflammation [8,9]. Wrinkles increase the demand for services that reduce visible aging and functional preservation of the skin. They interact with the aesthetic, psychological, and social domains [10,11]. Protecting skin from aging and environmental assaults is a multifaceted approach. It includes enhancing skin renewal, hydration, and anti-inflammatory, antioxidant, and protective barriers [12,13]. When a single agent may not achieve all desired results, combining multiple therapeutic approaches may be warranted. Consequently, the use of whole natural products with multiple active constituents is becoming more preferred [14,15]. They are better accepted by the body and safer than synthetic products [16]. Flavonoids, phenolic acids, and polyphenols are among many phytochemicals that improve skin health. Some bioactive skin-protective and anti-inflammatory compounds include salvianolic acids, rosmarinic acids, rosehips, niacinamide, and caffeine [16]. Plant-derived compounds rich in flavonoids, polyphenols, and phenolic acids significantly influence skin health. Caffeic acid, danshensu, resveratrol, nicotinamide, rosehips, rosmarinic acid, and salvianolic acid are bioactive compounds that benefit the skin by combating inflammation and providing protection [17,18].

Resveratrol (RSV), a naturally occurring polyphenolic compound, responds to environmental stressors [19]. RSV can be found in both the cis and trans isomer forms with only the trans being considered bioactive [20]. Food sources for trans-resveratrol include grapes, red wine, and berries, as well as peanuts and dark chocolate. RSV has the ability to activate the Nfr2/ARE, SIRT1, AMPK, and MAPK pathways while also exerting anti-inflammatory action through the inhibition of NF-κB. RSV increases the expression of protective and restorative skin enzymes and antioxidants, diminishes oxidative skin damage, and regulates skin damage through the modulation of tyrosinase activity and skin inflammatory mediators [19,20]. RSV has also been shown to markedly reduce sebum production through the inhibition of the sebum promoting SREBP-1 (sterol regulatory element-binding protein-1) and other lipogenic enzymes [21]. Topical formulations containing RSV have been shown to improve skin appearance and texture by reducing fine lines, wrinkles, and pigmentation and by improving the skin’s elasticity while also being well tolerated and safe. However, the majority of active compounds in clinical trials have been shown to contain RSV as one of multiple active compounds and leave a significant gap in the research of trans-resveratrol as a sole active compound [22,23].

Nicotinamide (NCT) is an NAD^+^ precursor involved in energy metabolism, management of oxidative stress, and activation of the sirtuin pathways [24,25]. Since UV-B-induced skin damage is associated with oxidative stress and inflammation, NCT plays an important role in protecting the skin from the effects of photoaging [26]. Previous studies have shown that NAD^+^ precursors, including nicotinamide, improve skin barrier function, skin hydration, and dermal collagen content [27,28]. Research on the photoprotective mechanisms of nicotinamide has used, for example, intraperitoneal injection, which is not practical, as they serve no purpose [29,30,31]. Consequently, developing effective topical delivery systems for nicotinamide is crucial to maximizing its dermatological benefits [32]. As such, it is crucial to optimize the design of effective topical delivery systems for nicotinamide to maximize its benefits [33,34]. The progress of contemporary nanotechnology has enabled the development of advanced drug delivery systems that are more targeted, have improved bioavailability, and are sustained for longer periods. Many researchers are focused on the development of advanced nanoformulations, including ufasomes and cubosomes, of the bioactive compounds resveratrol and nicotinamide to overcome the challenges of skin penetration, retention, and bioavailability [35,36]. A unique advantage is that MNs (microneedles) can remain on the skin for an extended period, improving the transcutaneous delivery of medicinal compounds. Nanostructured lipid carriers, solid lipid nanoparticles, liposomes, niosomes, ethosomes, invasomes, and nanofibers are all examples of commercially available technologies [12,37].

Cerosomes (CRs), tubular vesicles composed of ceramide and having skin lipid analogs [38,39], have recently been studied for possible application in skin treatment. They strengthen the epidermal barrier while improving the stability and effectiveness of drugs [40]. The stability, mechanical properties, and drug-release profiles of vesicles are improved by surfactants and polymeric stabilizers, such as poloxamers. Despite advances in topical formulations, the stratum corneum remains a barrier to efficient transdermal delivery [41,42].

Microneedle (MN) technology is widely regarded as a minimally invasive and typically painless transdermal delivery strategy, as it creates transient microchannels within the stratum corneum without stimulating deeper nociceptors associated with conventional hypodermic needles [43,44,45]. MNs are transdermal and layered injections that provide the best of both worlds, leading to the least discomfort and the highest patient compliance [46].

Nicotinamide (NCT) and Resveratrol (RSV) have distinct effects but act synergistically to mitigate damage caused by photoaged skin. RSV works with and against other signals to reduce damage. RSV also acts through SIRT1 and AMPK via Nrf2/ARE and MAPK to reduce and prevent inflammation and oxidative damage. RSV also helps keep the dermal matrix intact. Furthermore, NCT improves cellular NAD levels and mitochondrial redox, both of which are critically depleted by chronic UV exposure. Therefore, the combination of RSV with NCT is believed to improve most, if not all, processes damaged by UV. NCT helps restore NAD-dependent antioxidants and repair mechanisms. RSV directly causes disability and inflammatory damage. While this specific study was not intended to differentiate between additivity and synergy, it served as the basis for the RSV-NCT combination, along with other hypotheses.

The objective of the present work is to develop and evaluate a resveratrol/nicotinamide (RSV/NCT)-loaded cerosome system composed of phosphatidylcholine, ceramides (III, IIIB, and VI), and poloxamer surfactants, incorporated into PLGA-based microneedles for transdermal anti-aging therapy. The study encompasses formulation optimization, physicochemical characterization, morphological analysis, solid-state characterization, in vitro drug release, ex vivo permeation assessment, microneedle fabrication and mechanical evaluation, stability studies, and drug content analysis. Furthermore, the work includes in vivo evaluation using a UVA-induced skin-wrinkle mouse model, supported by macroscopic skin assessment, histopathological examination, Masson trichrome staining, biochemical analysis of inflammatory and oxidative stress markers, and gene expression studies related to skin regeneration and extracellular matrix remodeling.

## 2. Materials and Methods

### 2.1. Chemicals and Reagents

L-α-phosphatidylcholine (PC), Pluronic L121, and Pluronic P188 were purchased from Sigma-Aldrich (St. Louis, MO, USA). Ceramide III, ceramide IIIB, and IV were purchased from Evonik Co. (Essen, Germany). PLGA RG502 was purchased from Evonik Industries (Essen, Germany). PLGA has a characteristic viscosity of 0.16–0.24 dL/g and a Tg between 42–46 °C. Polyvinyl alcohol (PVA, 6.0 cp) was obtained from Shuangxin Environmental Protection Co., Ltd. (Beijing, China). Polyvinylpyrrolidone (PVP k90, MW 360,000 Da) was purchased from Xinkaiyuan Pharmaceutical Co., Ltd. (Xuchang, China). Chloroform and Methanol were procured from El-Nasr Pharmaceutical Chemicals Co. (Cairo, Egypt).

### 2.2. Quantitative High-Performance Liquid Chromatography Analysis of Resveratrol and Nicotinamide

Resveratrol and Nicotinamide were quantified using High-Performance Liquid Chromatography (HPLC) with a UV-Vis detector (Agilent 1260 Infinity II/DAD; Agilent Technologies, Santa Clara, CA, USA). Separation was done at room temperature with a C18 reverse-phase column (250 mm × 4.6 mm, 5 µm particle size). The mobile phase consisted of A, a mobile-phase gradient system of 1% MeOH + 0.1% H_3_PO_4_ (pH 2.0), and B, acetonitrile. It was used during analysis, with a runtime of 18 min. The mobile-phase ratio A/B was 55/45 (7 min) [47,48]. The column oven was set to 40 °C, and the flow rate was 1.0 mL/min under isocratic conditions. Chromatographic runs were performed at room temperature, with a total analysis time of 18 min per sample, including equilibration, gradient elution, and column reconditioning. For RSV, the detection wavelength was set at 307 nm, corresponding to its maximum absorbance, with linear calibration (R^2^ ≥ 0.999) over the range 2–10 μg/mL, in agreement with previously validated HPLC methods for resveratrol quantification. For NCT, detection was performed at 260 nm, with an analogous linear calibration range of 20–80 μg/mL and a correlation coefficient (R^2^) ≥ 0.999. To maintain sink conditions and prevent external auto-concentration, 300 μL aliquots were extracted at regular intervals and immediately replaced with pre-warmed phosphate-buffered saline (PBS, pH 7.4) [49,50]. Total RSV emissions were quantified over time using a standard calibration curve based on known concentrations.

### 2.3. Animals

The National Research Center in Cairo, Egypt, supplied thirty mature male Swiss Albino mice (23–30 g; 4–6 weeks old). After one week of acclimatization, mice were housed in polyethylene cages, six per cage, with a floor area of about 1500–1800 cm^2^. Throughout the experiment, they were kept in conventional polypropylene cages with four per cage and subjected to a proper temperature (25 ± 2 °C), humidity (60–70%), and light (12 h dark/light cycles). Mice were fed with conventional food and water. Animals were handled in accordance with the UK Animals (Scientific Procedures) Act 1986 and related standards, including the EU Directive 2010/63/EU on animal experiments. All animal handling and experimental procedures adhered to the Guidelines for the Care and Use of Laboratory Animals published by the National Institute of Health (NIH Publication No. 8023, revised 1978). The Research Ethics Committee of the Faculty of Pharmacy at Cairo University approved the research protocol under the approval number **PI 3882** on 28 April 2025.

### 2.4. Formulation and Characterization

#### 2.4.1. Development and Evaluation of Elastic RSV/NCT-Loaded Cerosomes (RSV/NCT-CRs)

**Figure 1:**
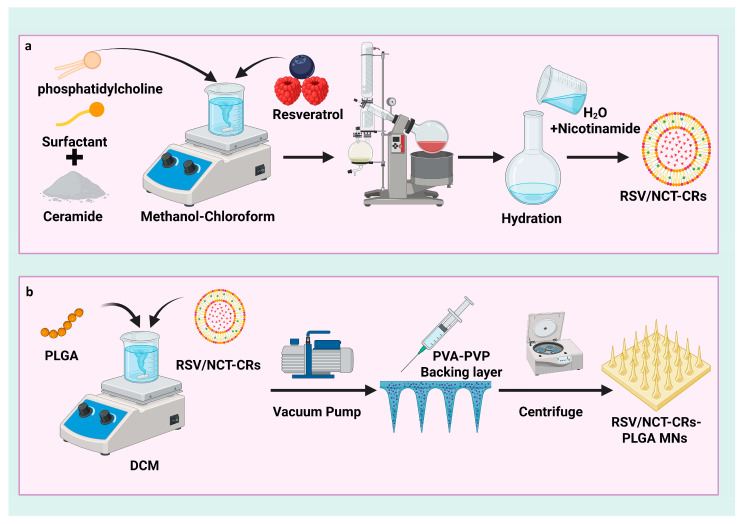
(**a**) Schematic diagram illustrates the methodology for preparing Resveratrol/Nicotinamide-Cerosomes (RSV/NCT-CRs), and (**b**) Schematic diagram illustrates the methodology for fabrication of multifunctional Optimized RSV/NCT-CRs-loaded PLGA microneedle patches (RSV/NCT-CRs/PLGA MNs).

Figure 1a and Table 1 illustrate the method and steps of formulating and preparing the RSV/NCT-CRs via the thin-film hydration method. Each of the three ceramides (ceramide III, ceramide IIIB, and ceramide VI) was administered at 25 mg. For each RSV/NCT-CRs formulation, phosphatidylcholine (PC) was incorporated at 50, 75, or 100 mg, together with 10 mg of resveratrol and 25 mg of a single ceramide subtype (ceramide III, IIIB, or VI) per batch as specified in the D-optimal design [39,41]. Each formulation contains 25 mg of Pluronic F127, F188, or L121 as a surfactant, with all components accurately measured and dissolved in a 10 mL methanol–chloroform solution (1:2, *v*/*v*). The solution was transferred to a round-bottom flask and rotary evaporated (Heidolph, Burladingen, Germany) at 60 °C and 90 rpm for 30 min under reduced pressure. The method resulted in the gradual evaporation of organic solvents, depositing a thin lipid layer on the interior wall of the flask [51,52]. The dry film was subsequently immersed in 10 mL of distilled water containing 10 mg of Nicotinamide at 60 °C, surpassing the temperature at which lipid phase changes transpire. The resultant dispersion was refrigerated overnight prior to additional characterization.

#### 2.4.2. Characterization of RSV/NCT-Loaded Cerosomes (RSV/NCT-CRs)

##### Entrapment Efficiency Percentage (EE%)

In the direct EE determination, the pelleted vesicular fraction was obtained after using an ultra-cooling centrifuge (Sigma 3-30 KS; Sigma Laborzentrifugen GmbH, Osterode am Harz, Germany). This (20,000 rpm, 60 min, 4 °C) was completely disrupted by re-dissolving in methanol, which solubilizes both lipid components and the entrapped drugs. This ensured the quantitative release of RSV and NCT from the cerosome bilayer prior to HPLC analysis, thereby allowing accurate calculation of the fraction of drug incorporated within the vesicle. The entrapped RSV and NCT concentrations were then measured using High-Performance Liquid Chromatography (HPLC) with a UV-Vis detector (Agilent 1260 Infinity II/DAD; Agilent Technologies, Santa Clara, CA, USA). The resulting pellet containing entrapped RSV and NCT was collected and dissolved in methanol to disrupt the vesicles and release the drugs prior to HPLC quantification (λ_max_ at 307 and 260 nm, respectively) [53,54]. All measurements were performed in triplicate, and mean ± SD values were caltriplicate an drug’s EE% was calculated using Equation (1):

(1) $$\mathrm{EE}\%\;\frac{Incorporated\;amount\;of\;RSV,or\;NCT}{Total\;amount\;of\;RSV\;or\;NCT}\times 100$$

All measurements were performed in triplicate.

##### Assessment of Particle Size and Zetapotential Measurement

A Nano ZetaSizer (Malvern Instruments Ltd., Malvern, UK) was utilized to evaluate the particle size (PS), polydispersity index (PDI), and zeta potential (ZP) of the synthesized RSV/NCT-CRs formulations. To attain a transparent and homogeneous suspension, the dispersions were appropriately diluted with distilled water (1:50) prior to measurements. The homogeneity of the vesicle size distribution was evaluated using PDI values, with higher values indicating greater variability and lower values indicating a more limited distribution. Based on the electrophoretic mobility of charged vesicles in an electric field, the ZP was determined to use the same device in conjunction with a zeta potential [1,2]. All measurements were performed in triplicate, and mean ± SD values were calculated (Table 2).

##### Statistical Optimization of RSV/NCT-CRs Formulations

The RSV/NCT-CRs formulations were optimized numerically utilizing Design-Expert 13^®^ software. The process was directed by specific criteria for the analyzed responses (%EE RSV, %EE NCT, PS, PDI, and ZP), aiming to enhance %EE and ZP while minimizing PS and PDI, as illustrated in Table 1. ANOVA was used to determine the effects and significance of the formulation components and their interactions on the measured responses. Each response was transformed into a desirability index using a desirability function. These indices were then combined into a single index ranging from 0 (undesirable) to 1 (most desirable). The formulation of RSV/NCT-CRs with the highest attraction value was selected as the preferred recipe for further study. The preferred formulation was re-optimized for %EE, PS, PDI, and ZP, and the resulting values were statistically compared with the predicted values to verify the process’s accuracy [55,56,57]. This refined nano dispersion was used as-is for all in vitro, ex vivo, and in vivo studies.

#### 2.4.3. In Vitro Evaluation of the Optimal RSV/NCT-CRs Formulation

##### Morphological Analysis Using Transmission Electron Microscopy (TEM)

The surface architecture of the improved RSV/NCT-CRs was analyzed using Transmission Electron Microscopy (TEM, model JEM-1230; JEOL, Tokyo, Japan) at an accelerating voltage of 80 kV. Prior to imaging, the mixture was diluted with deionized water to ensure adequate particle dispersion. A carbon-coated copper grid was meticulously coated with a small aliquot of the diluted sample, then air-dried at ambient temperature [58]. The grid was negatively stained with 2% (*w*/*v*) phosphotungstic acid to enhance picture contrast and render vesicular morphology more discernible. The resultant samples were analyzed by TEM after comprehensive drying.

##### Fourier-Transform Infrared Spectroscopy (FTIR)

FTIR spectra were obtained for RSV/NCT, optimized RSV/NCT-CRs, Blank-CRs, Blank PLGA, and RSV/NCT-CRs-PLGA MNs formula using a Bruker FTIR spectrophotometer (model 22, Coventry, UK). The analysis focused on possible interactions between ESV-NCT and other components of the prepared system. For each sample, 3 mg of anhydrous KBr was weighed, mixed, and pressed into a pellet. Each spectrum was recorded at room temperature over 4000–500 cm^−1^, with 32 scans per sample and a spectral resolution of 4 cm^−1^. The spectra were analyzed for the presence of distinct peaks corresponding to the sample components, and for possible increases, decreases, shifts, or changes in peak positions, which could signify interactions between the excipients and the drug [59,60].

##### Differential Scanning Calorimetry (DSC)

A DSC thermogram shows thermal characterizations using a Differential Scanning Calorimeter (DSC-60, Shimadzu Corporation, Kyoto, Japan) [calibrated with indium (m.p = 156.6 °C, 99.99% purity), heating rate = 10 °C/min]. Under a nitrogen gas flow of 20 mL/min, the samples (RSV/NCT, optimized RSV/NCT-CRs, Blank-CRs, Blank PLGA, and RSV/NCT-CRs-PLGA MNs) were analyzed within a temperature range of 30 to 450 °C in aluminum crimped pans [61,62].

##### Physical Stability Study

Stability of the optimized RSV/NCT-CRs was evaluated at 4 ± 3 °C and 25 ± 2 °C over 6 months, with samples analyzed at 0 (fresh), 1, 3, and 6 months for EE, particle size, PDI, zeta potential, and release profile. The samples were visually inspected for changes in physical properties, such as color, re-dispersibility, and the presence of visible aggregates, after being stored. Additionally, the primary physicochemical characteristics (%EE RSV, %EE NCT, PS, PDI, and ZP) were reassessed and compared with the freshly synthesized formulation [54,63]. Moreover, the release profiles of fresh and stored samples were compared by calculating the similarity factor (ƒ^2^) using Equation (2) below:

(2) $$f_{2}\;=50\;\times \;\log\;\{[1\;+\;(\frac{1}{n})\sum_{t=1}^{n}{\left(Rt-Tt\right)}^{2}{]}^{-0.5}\times 10$$

where n is the number of sampling points, and R_t_ and T_t_ are the cumulative percentage of medication released at time t from fresh and stored formulations, respectively. A value between 50 and 100 indicates that the two release profiles are close and exhibit similar release behavior after storage.

##### Freeze-Drying of Optimal RSV/NCT-CRs Formulation

Following loading of the CR dispersions with RSV and NCT, the dispersions were mixed with trehalose (5% *w*/*v*) as a cryoprotectant, rapidly frozen at −24 °C, and subsequently lyophilized in a Novalyphe-NL 500 lyophilizer at −24 °C and 7 mbar. Trehalose was selected based on its established ability to preserve the structural integrity of lipid vesicles during freeze-drying by forming a protective glassy matrix. Subsequent studies were conducted on the solid-state properties of the generated lyophilized CRs, including their components and physical composition [64,65].

#### 2.4.4. Design and Fabrication of Poly(lactic-co-glycolic acid) Microneedles (PLGA-MNs) Loading Freeze-Dried Optimal Resveratrol/Nicotinamide-Loaded Cerosomes (RSV/NCT-CRs)

To fabricate RSVNCT-CRs-loaded PLGA microneedles (PLGA-MNs), a two-step casting process was employed. In this hybrid design, the needle tips consist of a PLGA-rich phase containing freeze-dried RSV/NCT-CRs dispersed in PLGA solution in DCM, whereas the backing layer is formed from an aqueous PVA/PVP matrix, were sequentially cast onto a PDMS mold with a 10 × 10 array. The mold tip parameters (H300, B250, and P500); the mold tip parameters (height 300 μm, base width 250 μm, and pitch 500 μm) were selected to achieve homogeneous microneedle formation with sufficient length for skin insertion. The first casting solution was PLGA dissolved in DCM, lyophilized to optimal RSV/NCT-CRs, and 20 µL of pouring liquid was added to the mold. The vacuum pump creates a pressure of −100 kPa under the mold and maintains it for 10 min. After that, the mold with the solution was heated to 60 °C to shape the MN tips. The second phase, casting the baseplate, was performed after the tip layer had completely cured [66,67]. The baseplate was made by mixing PVP k90 and PVA with water, as described in Table 3. Rings were attached to the mold surface to secure the baseplate formulation, which was dispensed using a positive-displacement pipette and centrifuged at 3500 rpm for 10 min to fill the cavities. Next, introduce 70 μL of the second casting solution into the mold to form the backing layer. The mold with the backing layer solution was placed at 25 degrees Celsius and 30% relative humidity. The MNs were completely dry after 8 h. Peeling the MN patch off the mold included using a layer of medical tape, as shown in Figure 1b. The MN patch was removed from the mold using medical tape [68,69,70]. A blister pack consisting of PVC and aluminum was used to pack the Microneedle patch. The blister pack with the MN patch was packed in an aluminum-plastic bag, coupled with a bag of silica gel desiccant.

#### 2.4.5. Physical Characteristics of the RSV/NCT-CRs Loaded with PLGA Microneedles

##### Mechanical Testing and Penetration Capability Test

As previously stated [71], mechanical strength testing of the microneedle arrays was performed using the TA Electroforce 3200 system (TA Instruments, New Castle, DE, USA). To simulate skin implantation, arrays were taped to the moving crosshead using 3M double-sided tape. Compression was applied at a rate of 0.01 mm/s until a load of 32 N was achieved, held for 30 s, and then released at 0.1 mm/s. Load force and displacement were recorded at 20 Hz, and initial fracture force, stiffness, and holding displacement were calculated. The insertion and penetration characteristics were examined utilizing the previously validated Parafilm M^®^ model. Microneedle arrays were attached to the crosshead, and a stack of eight folded layers of 0.12 mm-thick Parafilm was positioned underneath. Insertion occurred at a rate of 0.1 mm/s until a load of 32 N was attained, maintained for 30 s, and thereafter released at 0.1 mm/s. The number of penetrations per layer was counted under a microscope using preset classification criteria. As previously reported, penetrations were classified as “holes, tears, or dents” based on the depth of puncture through the parafilm [72,73,74,75]. Equations (3) and (4) were used to calculate mechanical strength and penetration capability.

(3) $$\%\mathrm{Height}\;\mathrm{Reduction}=\frac{Initial\;height-Final\;heigh}{Initial\;height}\times 100\%$$

(4) $$\%\mathrm{Penetration}\;\mathrm{capability}=\frac{Number\;of\;holes}{Total\;needles}\times 100$$

##### Studies on Drug Content

An array of RSV/NCT-CRs-loaded PLGA-MNs magnetic agitators (IKA, Staufen, Germany) was dissolved in distilled water containing 2.5% Tween 80 for 1 h while stirred at 300 revolutions per minute. The resultant solution was diluted with methanol and sonicated for five minutes to fully dissolve the RSV/NCT from the manufactured RSV/NCT-CRs-loaded PLGA-MNs. The drug content of the MNs produced was determined by HPLC analysis(Agilent 1260 Infinity II/DAD; Agilent Technologies, Santa Clara, CA, USA), as previously stated [3,4,5].

##### Water Loss on Drying (LOD)

To determine the loss on drying (LOD), 0.5 g of the microneedle formulations were accurately weighed, dried in a desiccator for 48 h, and re-weighed [6,7]. The data from these measurements were analyzed using Equation (5):

(5) $$\%\mathrm{LOD}\;=\;\frac{Initial\;weight-Final\;weight}{Initial\;weight}$$

#### 2.4.6. Characterization of Optimized Microneedle

##### Scanning Electron Microscopy (SEM)

The morphology of the RSV/NCT-CRs-loaded PLGA-MNs patch was investigated using a scanning electron microscope (SU8010, HITACHI, Tokyo, Japan). The RSV/NCT patch was affixed to a microscope carrier, and visualization was conducted at 6 Kv with a magnification of 150× [8,9].

##### Differential Scanning Calorimetry (DSC)

The thermal properties, crystallinity, and interactions among Blank PLGA MNs, and RSV/NCT-CRs-loaded PLGA-MNs were examined using differential scanning calorimetry (DSC, Shimadzu Corporation, Kyoto, Japan), at temperatures ranging from 25 to 250 °C, utilizing nitrogen as the purging gas at a flow rate of 100 mL/min as mentioned before.

##### Fourier Transform Infrared (FTIR) Analysis

An FTIR spectrometer was used to record the room temperature FTIR spectra of a Blank PLGA MNs, and RSV/NCT-CRs-loaded PLGA-MNs patch and its constituent parts between 500 and 4000 cm^−1^ (Tensor II, Bruker, Ettlingen, Germany).

##### Drug Release Studies

- In vitro Drug Release Study

The in vitro release of RSV from REV suspension, RSV/NCT-CRs, and RSV/NCT-CRs-PLGA MNs, NCT from NCT solution, RSV/NCT from RSV/NCT-CRs, and RSV/NCT-CRs-PLGA MNs was measured using a USP dissolution testing apparatus type II (Hanson Research Corp., Agoura Hills, CA, USA). The dissolution media consisted of 100 mL of phosphate buffer (pH 6.8) containing 25% methanol to ensure sink conditions. The formulations (10 mg of RSV and NCT) were placed in two open-ended tubes, one sealed with a cellulose membrane and the other attached to the shaft of the dissolution device. The dissolution media was maintained at 37 ± 1 °C, with the shaft rotating at 50 rpm. At specific time intervals (0.25, 0.5, 1, 2, 4, 6, 8, 10, 12, 24 h), 1 mL samples from the dissolution media were removed and replaced with an equal volume of fresh medium. To elucidate the release mechanism, the release data obtained were fitted to the zero-order (Equation (6)), first-order (Equation (7)), Higuchi (Equation (8)), and Korsmeyer-Peppas (Equation (9)) models, which were used to describe the drug release mechanism [10,11]. The model providing the best fit was determined according to the highest correlation coefficient (R^2^). The following are the equations of different models:

The equations below show the mathematical models that were used:

Zero order:

Qn = Q0 + k0t(6)

First-order kinetics:

(7) $$\mathrm{Log}\;(100\;-\;\mathrm{Qn})\;=\;\log\;Q0\;-\;\frac{Kt}{2.303}$$

Higuchi diffusion:

Qn = kH√ t(8)

Korsmeyer-Peppas:

Log Qn = logk + nlogt (9)

Qt represents the amount of medication dissolved in time t, k: release constant, f is the fraction of PD released after the time (t), n: diffusional exponent (measuring the release mechanism), and β: describes a shape parameter (a measure of the releasing mechanism).

The coefficient of determination (R^2^) values from each model were compared, and the one with the greatest value (R^2^) was chosen as the best-fitting model to explain the PD release kinetics. The n and β factors are critical for determining the type of diffusion described by Fick’s equation. Fickian diffusion occurs when n ≤ 0.5 and β ≤ 0.75.

- Ex vivo permeation study

Ex vivo permeation assessments were performed utilizing a modified Franz diffusion cell. The membrane of the chicken pouch was affixed to both the donor and receptor compartments and was designed to emulate human buccal mucosa. Ten milligrams of each RSV from the RSV solution, NCT from the NCT solution, RSV/NCT from the RSV/NCT-CRs, and RSV/NCT-CRs -PLGA MNs (corresponding to 10 mg of active compounds) were accurately measured and placed into the donor compartment. The receptor compartment was filled with 25 milliliters of a pH 6.8 phosphate-buffered solution containing 25% methanol. This would help to achieve sink conditions. The sample was kept spinning magnetically at 100 rpm and 37 ± 1 °C. Every hour for twenty-four hours, one milliliter of the permeation medium was removed from the receiving cell and replaced with an equal volume of fresh medium. There were three replicate experiments. After filtering over a 0.45 µm membrane, the samples underwent validation HPLC analysis. Time was plotted against the NCT, RSV concentration that went through the Fresh Chicken Pouches membrane. The apparent skin permeability coefficient (cm/h) was calculated using the equation Kp = Jss/C_0_, where C_0_ was the starting drug concentration (µg/h·cm^2^), and Jss (steady state flux) was the slope of the linear part (µg/cm^2^). After carefully cleaning the Fresh chicken pouch membranes three times with deionized water, the membranes were soaked overnight in 20 mL of methanol, then sonicated for 15 min in a sonicator bath at the end of the ex vivo permeation experiment [12,13]. Samples were collected, passed through a 0.45 µm membrane, and analyzed using a validated HPLC technique to determine the amount of RSV and NCT deposited in the mucus tissue after 24 h.

### 2.5. In Vivo Studies

#### 2.5.1. Groups and Induction of Wrinkles

The mice’s dorsal fur was depilated with shaving foam, and the depilated area was irradiated with UVA irradiation using a UVA lamp emitting predominantly in the 320–400 nm range with a peak at 365 nm (irradiance 10 ± 1 mW/cm^2^ at the dorsal skin surface). Mice in groups GII–GV were exposed three times per week for 8 consecutive weeks, with each session lasting 30 min, corresponding to a per-session dose of approximately 18 J/cm^2^ and a cumulative dose of ~432 J/cm^2^ over the study period. During irradiation, non-dorsal areas were shielded to restrict exposure to the target. The mice were positioned 15 cm from the lamp (Sylvania^®^ Blacklight Blue fluorescent lamp, F20W/T12/BL, 368.20 W; Erlangen, Germany) [76,77]. Wrinkles were elicited, and therapy was administered continuously for 14 days. The mice were randomly grouped into five groups as follows: (6 mice/group)

The First group (GpI) (healthy control mice (GI) had not undergone UVA radiation exposure and served as a baseline for normal skin.

The second group (GpII) (positive control, untreated mice): these mice exhibited wrinkle formation following UVA irradiation without treatment; these subjects served as a baseline for comparison [77].

The third group (GpIII) (wrinkled mice treated with RSV/NCT (2% *w*/*w* HPMC) gel: these mice developed wrinkles following UVA light exposure and were administered RSV/NCT gel for daily application throughout the treatment cycle (14 days).

The fourth group (GPIV) (wrinkled mice received treatment with RSV/NCT-CRs (2% *w*/*w* HPMC) gel: these mice developed wrinkles and were administered an RSV/NCT-CRs gel daily for 14 days.

**Figure 2:**
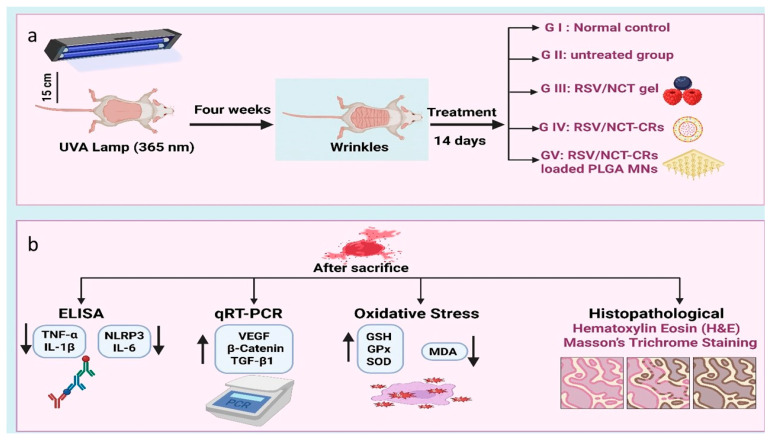
Experimental design and evaluation workflow of in vivo UVA-induced skin wrinkles in mice. (**a**) In vivo treatment and grouping (**b**) Biochemical and histopathological studies after sacrificing.

The fifth group (G V) (wrinkled mice received treatment with the RSV/NCT-CRs-PLGA MNs patch): these mice developed wrinkles by exposure to a UVA lamp and received treatment with a microneedle patch. This grouping strategy allowed stepwise comparison of (i) free drug in a conventional topical vehicle, (ii) nanocarrier-based cerosomal encapsulation, and (iii) cerosomes combined with minimally invasive microneedle-assisted delivery. The treatment was applied to the wrinkled skin once, then a new patch was applied daily for 14 days, as shown in Figure 2.

#### 2.5.2. Animal Sacrifice and Tissue Collection

Animals were anesthetized with ketamine (100 mg/kg) and xylazine (20 mg/kg) prior to euthanasia in accordance with established methods. A 1 cm^2^ (1 cm × 1 cm) section of dorsal skin was excised using a surgical scalpel and scissors. About 50 mg of skin tissue was collected and completely submerged in RNA later reagent (Qiagen, Hilden, Germany). It was then incubated at −80 °C for subsequent mRNA quantification. Some of the samples were preserved in 5% formalin for histological examination. Frozen dermal specimens were homogenized for biochemical analysis. In summary, 100 mg of skin tissue was homogenized in 1 mL of ice-cold phosphate-buffered saline (PBS, pH 7.4). The homogenate was centrifuged at 15,000 rpm for 20 min at 4 °C, and the supernatant was transferred into three sterile microtubes and stored at −80 °C for subsequent investigation of inflammatory, enzymatic, and oxidative stress markers.

#### 2.5.3. Enzyme-Linked Immunosorbent Assay (ELISA) for Inflammatory Markers

The concentrations of pro-inflammatory cytokines in skin homogenates, specifically tumor necrosis factor-alpha (TNF-α), leucine-rich repeat and pyrin domain-containing protein 3 (NLRP3), interleukin-1 beta (IL-1β), and interleukin-6 (IL-6) were measured using commercially available ELISA kits (LifeSpan Biosciences, Inc., Seattle, WA, USA) based on the sandwich principle. The absorbance was assessed spectrophotometrically at 450 nm, following the manufacturer’s instructions [2,78,79].

#### 2.5.4. Quantitative Real-Time Polymerase Chain Reaction (qRT-PCR)

Total RNA was extracted from homogenized tissues of all groups using the QIAzol reagent (Qiagen, Germany) according to the manufacturer’s protocol, and the quantity and quality of the RNA yield were assessed using a Nanodrop (Thermo Fisher Scientific, USA). Reverse transcription of 1 μg RNA into cDNA was performed according to the manufacturer’s instructions of the SensiFAST™ cDNA Synthesis Kit (Bioline, London, UK). Finally, cDNA templates were amplified using a real-time PCR apparatus (Applied Biosystems 7500, USA) with the following amplification profile: 2 min at 95 °C, followed by 40 cycles of 10 s at 95 °C and 30 s at 60 °C [80,81]. The amplification reaction contained 10 μL of HERA SYBR green PCR Master Mix (Willowfort, Nottingham, UK), 2 μL of cDNA, 1 µL of forward primer, 1 µL of reverse primer, and 6 μL of nuclease-free water, for a total reaction volume of 20 µL [18,19]. The relative quantitation (RQ) of mRNA expression for VEGF, TGF-β1, and β-catenin was calculated using the 2^−∆∆Ct^ method [20]. All gene expression values were standardized using glyceraldehyde-3-phosphate dehydrogenase (GAPDH) as the housekeeping reference gene. The specific primers are listed in Table 4. Primers were designed using Primer3 software (v. 4.1.0; http://primer3.ut.ee). The Primer-BLAST (https://www.ncbi.nlm.nih.gov/tools/primer-blast/, accessed on 5 February 2026.) software was used to assess primer specificity, and melting curve analysis was performed [21,82].

#### 2.5.5. Effect of Treatments on Oxidative Stress and Antioxidant Defense Markers

##### Malondialdehyde (MDA) Analysis

Malondialdehyde (MDA) levels in skin homogenates were quantified using the thiobarbituric acid reactive substances (TBARS) assay (Biodiagnostic, Cairo, Egypt) as per the manufacturer’s instructions. This method employs the interaction between MDA, a terminal byproduct of lipid peroxidation from unsaturated fatty acids, and 2-thiobarbituric acid (TBA), resulting in a pink chromogenic adduct. In the experiment, 300 µL of 2-thiobarbituric acid solution was combined with 50 µL of each sample, and the mixture was incubated in a water bath at 90 °C for 30 min. The concentration of MDA (Biodiagnostic, Egypt) was determined by measuring absorbance at 532 nm with a microplate reader after adding 100 µL of each sample to the wells. The absorbance of the solution is directly proportional to the extent of lipid peroxidation [83,84].

##### Measurement of Reduced Glutathione (GSH)

Reduced glutathione (GSH) levels in skin tissue homogenates were quantified using a commercially available colorimetric assay kit (Biodiagnostic, Egypt) based on reaction with Ellman’s reagent (5,5′-dithiobis-2-nitrobenzoic acid), following the manufacturer’s protocol. After incubation with the reagent, absorbance was measured at 412 nm using a microplate reader, and GSH concentrations were calculated from the standard curve and expressed as μmol/g tissue [85,86].

##### Measurement of Glutathione Peroxidase (GPx)

Glutathione peroxidase (GPx) activity in skin tissue homogenates was determined using a colorimetric GPx assay kit (Biodiagnostic, Egypt) according to the manufacturer’s instructions. The assay monitors the rate of oxidation of reduced glutathione in the presence of hydroperoxides, generating a chromogenic product. Absorbance was recorded at the specified wavelength with a microplate reader, and GPx activity was expressed as U/g tissue [87].

##### Assessment of Superoxide Dismutase (SOD)

Superoxide dismutase (SOD) activity in skin tissue homogenates was measured using a colorimetric SOD assay kit (Biodiagnostic, Egypt) in accordance with the manufacturer’s instructions. The method is based on inhibition of the reduction in a chromogenic substrate by superoxide radicals. Absorbance was read with a microplate reader at the recommended wavelength, and SOD activity was expressed as U/g tissue [87,88].

### 2.6. Histopathological Evaluation Using Hematoxylin Eosin (H&E) and Masson’s Trichrome Staining

After at least 24 h of fixation, skin samples were dehydrated using graded ethanol, cleared in xylene, and embedded in paraffin. A Leica RM2145 microtome was used to section paraffin blocks into 5–6 µm slices. The sections were mounted on slides and stained with hematoxylin and eosin (H&E) and Masson [89,90]. HE staining was used to monitor changes in epidermal and dermal structures, and epidermal thickness was assessed with ImageJ software (National Institutes of Health, Bethesda, MD, USA; version 1.53), while Masson staining was used to assess collagen fiber damage. Histological investigation was carried out with a light microscope (CX41, Olympus, Tokyo, Japan) and a digital camera (DP25, Olympus) [25,91].

### 2.7. Statistical Analysis

The data was shown as the mean ± SD. Analysis of variance (ANOVA) was used to assess group differences, followed by Tukey’s post hoc test. In addition, an unpaired *t*-test was employed to compare differences between any two groups as necessary. Statistical significance was determined at *p* < 0.05 using version 9.2.2. GraphPad Prism^®^ software suite (GraphPad Software, San Diego, CA, USA).

## 3. Results and Discussion

### 3.1. Analysis of D-Optimal Design

A D-optimal factorial design was employed to systematically examine the effects of formulation variables PC quantity (X1), ceramide type (X2), and surface-active agent (SAA) type (X3) on the critical quality attributes of RSV/NCT-loaded cerosomes, specifically RSV entrapment efficiency (Y1), NCT entrapment efficiency (Y2), particle size (Y3), polydispersity index (Y4), and zeta potential (Y5), as detailed in Table 1. The methodology used in this study enabled rapid mapping of the formulation space while minimizing the number of experiments and identifying both the main and interaction effects related to vesicle function.

#### 3.1.1. Effect of Formulation Variables on Entrapment Efficiency (EE%)

Across the various formulations, the entrapment efficiency of RSV and NCT showed the most variability. RSV EE% ranged between 48 and 91% and NCT EE% ranged between 44 and 85% (Table 2). The statistical analysis showed that the EE% models were highly significant (Table 5). Responding to the formulation of PC (A) and SAA type (C) was the main contributor to RSV entrapment. In terms of NCT entrapment, SAA type (C) was the most significant.

##### Effect of PC Amount (X1)

**Figure 3:**
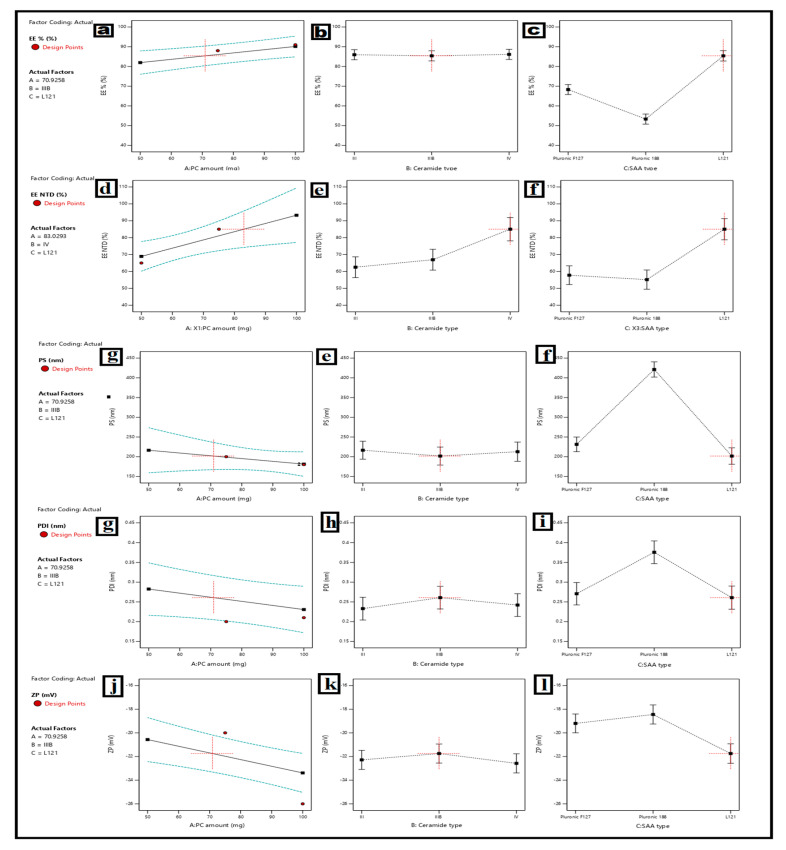
Effect of formulation variables on the physicochemical properties of RSV/NCT-loaded cerosomes as analyzed by the D-optimal design. Main-effect and interaction plots illustrating the influence of PC amount (X1), ceramide type (X2), and surface-active agent (SAA) type (X3) on (**a**–**c**) entrapment efficiency of RSV (Y1), (**d**–**f**) entrapment efficiency of NCT (Y2), (**g**–**i**) particle size (Y3), (**j**–**l**) polydispersity index (Y4), and (**j**–**i**) zeta potential (Y5).

After comparing the main-effect plots (Figure 3), increasing PC dosage from 50 mg to 100 mg has improved EE% for both medicines. Among the formulations of PC 100 mg, in particular Runs 1 and 2, achieved RSV EE% exceeding 87%. This can be attributed to the formation of a lipid bilayer that is both more rigid and viscous. A greater concentration of phospholipids means a greater degree of restriction in the diffusion of the drug from the vesicular core, and a decrease in leakage during the processes of preparation and storage [35,92]. This was crucial for RSV because it is a highly lipophobic molecule, and the greater the bilayer rigidity, the greater the lipophilic dominance in the lipid domain.

##### Effect of Ceramide Type (X2)

The type of ceramide has a moderate, consistent effect on EE%. Among the three ceramides, IIIB had the highest EE% compared to III and IV. This is clear in the contrast of formulations with the same levels of PC and SAA (e.g., Runs 1 vs. 2 and Runs 14 vs. 12). Ceramide IIIB most likely promotes RSV incorporation and sufficient encapsulation of the relatively hydrophilic NCT, thereby compromising lipid structure and the NCT’s hydrophobic entrapment within the aligned lipid membrane [92].

##### Effect of SAA Type (X3)

Formulations made with Pluronic L121 achieved the highest EE% for both RSV and NCT (e.g., Runs 1, 12, 14, and 17), indicating that Pluronic L121, as an SAA, positively impacted drug entrapment. Pluronic F127 and Pluronic 188 were consistently outperformed. The main reason Pluronic L121 shows improvement is its greater hydrophobicity and lower HLB. This means there is tighter packing of the membrane, resulting in a constriction of drug transport across the vesicular bilayer. In contrast, the more hydrophilic Pluronic 188 had a lower EE%; a potential cause being greater membrane permeability and drug loss [92,93]. The interaction graphs (Figure 3) show a prominent synergistic effect of high levels of PC and Pluronic L121 as SAA, resulting in the best drug entrapment. This shows that the best EE% is due to the combination of the high PC concentration and hydrophobic SAA, creating a synergistic effect in the vesicle’s interior.

#### 3.1.2. Effect on Particle Size (PS) and Polydispersity Index (PDI)

Particle sizes were recorded between 180 and 430 nm, and PDI were 0.20–0.44 (Table 2). The statistical models were significant for PS and PDI, with PC amount (A), ceramide type (B), and SAA type (C) all size modulators (Table 5).

##### Effect of PC Amount

An increase in PC amount resulted in smaller, more homogeneous vehicles, particularly at moderate levels (75 mg). This indicates that self-assembly and membrane integrity are optimal at moderate levels of lipids, even though the sizes of the vesicles formed are relatively larger, and more complex vesicle structures, because bilayer dehydration cannot be formed, with 50 mg of phosphatidylcholine (refer to Runs 21 and 6).

##### Effect of Ceramide and SAA Types

L121-containing formulations showed consistently lower PS and PDI, implying better vesicle stability and lower aggregation. On the other hand, Pluronic 188 produced larger, more polydisperse systems, especially at lower PC concentrations, which can be ascribed to weaker hydrophobic interactions and greater vesicle fusion. Smaller PDI values (~0.20–0.21) observed with 75–100 mg of PC and Pluronic L121 formulations indicate a highly monodisperse population, which is desirable for topical and transdermal applications.

#### 3.1.3. Effect on Zeta Potential (ZP) and Vesicle Stability

The vesicular systems were electrostatically stable, with ZP values ranging from −17 to −26 mV across the formulations. Table 4 indicates the effects of SAA type and amount of PC on ZP. ZP values were markedly lower with Pluronic L121 combinations. In formulations containing Pluronic L121, more negative ZP values indicate better colloidal stability and a higher surface charge density. In contrast, Pluronic 188-based systems agglomerate more during storage due to their higher ZP values, leading to reduced stability. Based on the data received, most processed formulations produced ZP values of 20 mV or higher, indicating stable colloidal systems.

#### 3.1.4. Optimization and Selection of the Optimal Formula

Although several Pluronic L121–containing formulations showed high entrapment efficiency and acceptable size, Run 14 (75 mg PC, ceramide IIIB, Pluronic L121) achieved the highest overall desirability score because it offered the best compromise among all critical quality attributes. Compared to other L121 formulations, Run 14 combined high RSV and NCT entrapment (≈88% and ≈63%, respectively) with a small mean particle size (~200 nm), low PDI (~0.20), and sufficiently negative zeta potential (~−20 mV). Formulations with higher PC (100 mg) or alternative ceramides tended to produce larger or more polydisperse vesicles, which can negatively affect skin penetration and physical stability. Thus, Run 14 was selected as the optimal formulation because it maximized simultaneous fulfillment of loading, size, homogeneity, and surface charge criteria rather than excelling in a single parameter alone [39,93,94,95].

### 3.2. Morphological and Physicochemical Characterization

#### 3.2.1. Transmission Electron Microscopy (TEM)

**Figure 4:**
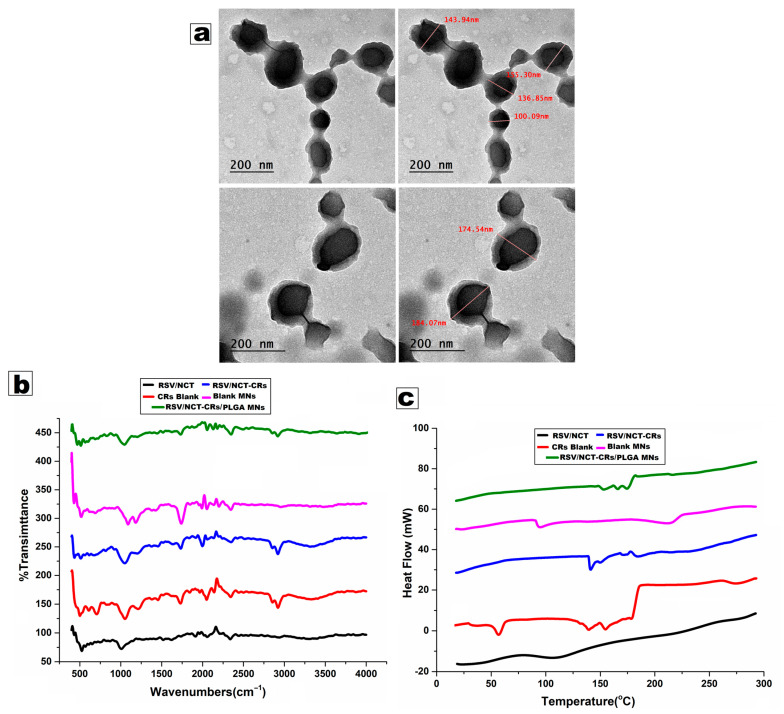
(**a**) TEM micrograph of optimized Resvatrol/Nicotinamide–Cerosomes (RSV/NCT-CRs), (**b**) Fourier Transform Infrared Spectroscopy, and (**c**) Differential Scanning Calorimetry for pure Resvatrol/Nicotinamide (RSV/NCT), Resvatrol/Nicotinamide–Cerosomes (RSV/NCT-CRs), blank Cerosomes (Blank-cerosomes), Blank PLGA-MNs (Blank-MNs), and RSV/NCT-CRs-loaded PLGA MNs.

TEM micrographs (Figure 4a) showed that the optimized RSV/NCT-loaded cerosomes displayed well-defined nanovesicular structures, characterized by predominantly non-spherical morphologies, including elongated and tubular-shaped vesicles, with particle sizes aligning with DLS measurements (≈100–180 nm). The noted divergence from traditional spherical vesicles can be attributed to the inclusion of ceramide in the phosphatidylcholine (PC) bilayer, which significantly alters membrane curvature and packing dynamics. This has been observed in cerosome systems, where the addition of ceramides to PC bilayers has been attributed to increased membrane rigidity and reduced bilayer curvature, leading to elongation of vesicles into a tubular structure. Ceramides have a considerably higher packing parameter of roughly 1.2, compared to the 0.7 of phosphatidylcholine, leading to a bilayer that is less curved and more planar. The asymmetric distribution of ceramides in the bilayer membrane explains the formation of both ceramide-rich flattened vesicles and ceramide-poor spherical vesicles, a phenomenon previously described. The presence of these tubular and bulbous nanostructures is advantageous for topical and transdermal delivery, as these morphologies are associated with improved skin adhesion and prolonged drug retention in the stratum corneum.

#### 3.2.2. Fourier Transform Infrared (FTIR) Spectroscopy

FTIR Spectroscopy (Figure 4b) was conducted to assess the compatibility of the drugs and excipients and the types of molecular interactions present in the systems. The pure RSV showed distinct absorption bands in the 3200–3400 cm^−1^ range, corresponding to O-H stretching. The bands observed in 1605–1620 cm^−1^, and 1510 cm^−1^ are attributed to the C=C stretching of the aromatic and phenyl ring, respectively. Pure NCT exhibited significant peaks at (3300–3400 cm^−1^) (N-H stretching), (1650–1670 cm^−1^) (amide C=O stretching), and (1280–1320 cm^−1^) (C-N stretching). Typical peaks were observed in RSV/NCT-loaded cerosomes, but with slight changes and lower intensities. The O-H/N-H stretching band at ~3300 cm^−1^ became wider, while the amide carbonyl peak shifted slightly to ~1640–1650 cm^−1^. The observed changes suggest that RSV and NCT were most likely contained within the lipid bilayer via hydrogen bonding and hydrophobic interactions, rather than chemical destruction. PLGA microneedles exhibited a characteristic ester carbonyl stretching band at 1750–1760 cm^−1^ and C–O–C stretching bands between 1080 and 1180 cm^−1^, confirming the integrity of the PLGA backbone after microneedle fabrication. The presence of these peaks in RSV/NCT-CRs-loaded PLGA MNs, with the retention of drug-related bands, indicates that the processes of drug encapsulation and microneedle production were chemically compatible. The absence of new peaks across all formulations indicates no covalent interactions or chemical instability.

#### 3.2.3. Differential Scanning Calorimetry (DSC)

The Thermograms from differential scanning calorimetry (DSC) (Figure 4c) provides further insight into the physical state of RSV and NCT in the formulations. The pure RSV exhibited a clear endothermic melting peak near (253–255 °C), while pure NCT showed a characteristic melting endotherm at approximately (128–130 °C), thus confirming their crystalline characteristics. On the contrary, the individual melting peaks changed position, or, were absent in the RSV/NCT-loaded cerosomes. In contrast, wider and gentler thermal transitions were observed, which may suggest that both pharmaceuticals were present in an amorphous or molecularly dispersed state within the cerosomal lipid matrix. This is due to the strong interrelation of the drugs and lipids, as well as the ceramide-induced rigidity of the bilayer, which restrains the crystallization of the drugs. When the RSV/NCT-CRs were loaded into the PLGA microneedles, the melting peaks of RSV and NCT disappeared, and a broad glass transition and thermal event of the PLGA matrix were observed at 45–60 °C, a common occurrence for this polymer. The absence of crystallized drug-related endotherms in this system confirms successful drug entrapment in both cerosomes and microneedles, thereby ensuring greater physical stability and enhanced bioavailability.

#### 3.2.4. Stability Study of Optimized RSV/NCT-Loaded Cerosomes

The physical stability of the optimized RSV/NCT-loaded cerosomes (RSV/NCT-CRs) was studied for 6 months at refrigerated (5 ± 3 °C) and ambient (room) temperatures (Table 6). During 6 months of storage, RSV and NCT entrapment efficiencies showed only modest decreases (≤8–9% absolute loss), while particle size and PDI remained within the nanoscale and narrow distribution ranges, and zeta potential stayed more negative than −20 mV. These findings indicate that the ceramide-enriched vesicles maintain both colloidal stability and high drug retention over the tested period. From a translational standpoint, such limited changes are unlikely to compromise topical performance and suggest that refrigerated storage can provide a practical shelf-life for the optimized RSV/NCT-CRs formulation. RSV processing and NCT, EE % under refrigerated conditions decreased from 91% to 85% and from 85% to 79%, respectively, while in ambient conditions the percentages also decreased to 83% and 76%, respectively. The Phase transition closure and bilayer rearrangement increased drug retention, while the subtle reductions demonstrated the efficacious nature of ceramide-based packing. The increase in PDI and particle size was minimal, and the storage conditions are well accepted at the nanoscale. The PS of the vesicles was below 209 nm, and the PDI was below 0.35, indicating that there was no significant aggregation and that the uniformity of the vesicular population was maintained. The zeta potential was below −20 mV throughout the experiment, indicating sufficient colloidal stability and responsive electrostatic stabilization under both storage conditions. The statistical analyses confirmed that there were no significant differences across all evaluated parameters between the newly synthesized samples and those that had undergone storage. Moreover, comparison of the in vitro release profiles of freshly prepared versus stored RSV–NCT-CRs revealed f^2^ similarity factors above 50 for both drugs under both storage conditions, indicating that the release mechanism and kinetics were conserved during the 6-month period. Over time, the RSV/NCT-CRs demonstrated even greater stability in all evaluated factors. While refrigeration improved entrapment efficiency and particle uniformity, room-temperature stability was shown to be sufficient.

### 3.3. Physical Characteristics of the RSV/NCT-CRs Loaded with PLGA Microneedles

#### 3.3.1. Mechanical Strength and Height Reduction

**Figure 5:**
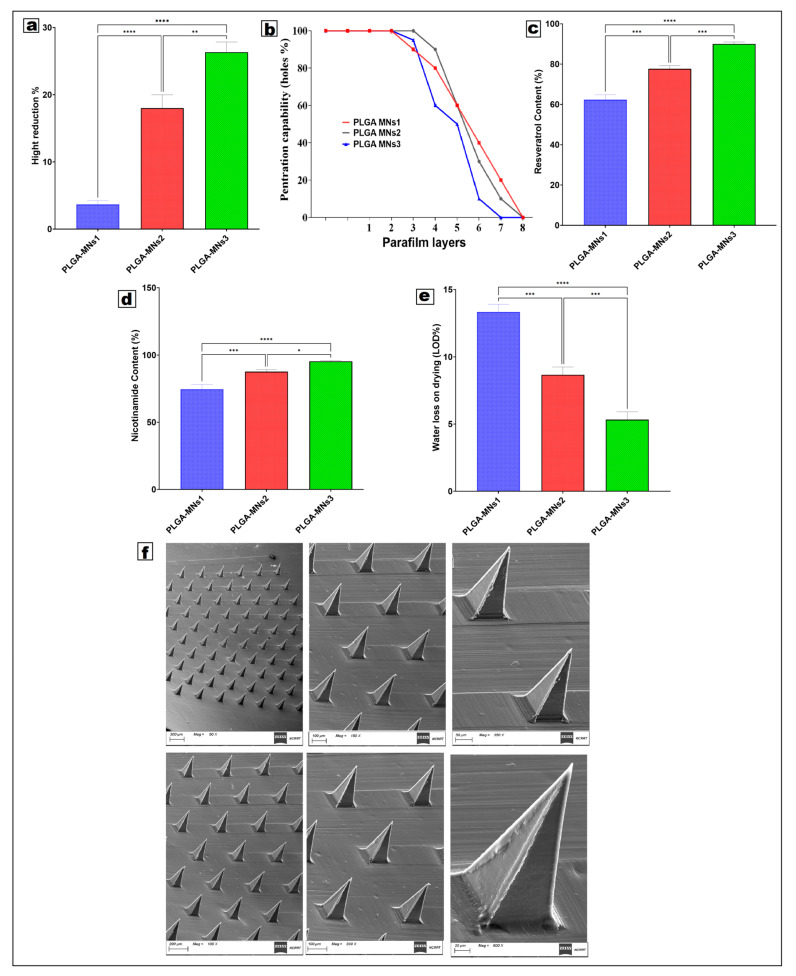
(**a**) Percentage height reduction in PLGA microneedles (PLGA-MNs 1–3), (**b**) Penetration capability (%) of PLGA-MNs, (**c**) Drug content (%) of nicotinamide (NCT) incorporated within RSV/NCT-CRs loaded PLGA-MNs, (**d**) Drug content (%) of Resveratrol (RSV) incorporated within RSV/NCT-CRs loaded PLGA-MNs, (**e**) Water loss on drying, and (**f**) Scanning electron microscopy (SEM) images of RSV/NCT-CRs loaded PLGA-MNs. Data are presented as mean ± SD (n = 3). Statistical significance was analyzed using one-way ANOVA followed by post hoc comparisons; * *p* < 0.05, ** *p* < 0.01, *** *p* < 0.001, and **** *p* < 0.0001.

As depicted in Figure 5a, the percentage reduction in height decreased with increasing PLGA concentration. PLGA-MNs 3 (10% *w*/*w* PLGA) demonstrated the least height drop, signifying enhanced resistance to axial compression, while PLGA-MNs 1 (2.5% *w*/*w* PLGA) exhibited the greatest deformation. This behavior results from heightened polymer chain entanglement and matrix rigidity associated with elevated PLGA content, which significantly increases needle stiffness and diminishes plastic deformation during compression. Conversely, formulations with reduced PLGA and elevated hydrophilic polymer ratios exhibited a greater susceptibility to elastic deformation and partial failure under stress.

#### 3.3.2. Penetration Capability and Insertion Performance

The data regarding penetration efficiency (Figure 5b) showed dependence on formulation composition. PLGA-MNs 3, for instance, showed the most notable penetration characteristics, creating a higher percentage of holes across multiple layers of Parafilm^®^. Following this were PLGA-MNs 2 and PLGA-MNs 1. The gradual reduction in penetration across multiple layers of Parafilm suggests a consistent loss of energy with continued insertion and supports the mechanical hierarchy among the formulations. The enhanced insertion efficacy of PLGA-MN 3 can primarily be attributed to its higher fracture force and stiffness, which enable it to puncture a stratum corneum-mimicking layer without significant blunt-tip fracture. In contrast, PLGA-MNs 1 demonstrated premature failure in penetration, attributed to the high levels of plasticization induced by PVA and PVP, leading to inadequate retention of tip sharpness under load. These results corroborated with prior studies that utilized Parafilm^®^ models to predict the insertion behavior of the skin.

#### 3.3.3. Drug Content Uniformity

In the analysis of drug content (Figure 5c,d), it was observed that, as PLGA concentration increased, the retention of RSV/NCT within the microneedle matrix increased. Among the three formulations, PLGA-MNs 3 had the most drug content, while PLGA-MNs 1 had the least. The improved encapsulation efficiency of tightly packed PLGA networks results in less drug diffusion during curing and solvent evaporation. The stability of the drug and the uniform dispersion achieved by incorporating freeze-dried cerosomes into the PLGA matrix enabled effective dual-drug loading.

#### 3.3.4. Water Loss on Drying (LOD)

PLGA content affected LOD (Figure 5e). PLGA-MNs 1 had the highest LOD, which can be attributed to increased moisture retention from the PVA’s hydrophilic properties. In contrast, PLGA-MNs 3 had a lower LOD, which can be attributed to the balanced formulation’s enhanced stability and dryness. The increased dryness improved storage longevity and mechanical strength, and safeguarded the formulation against drug degradation, especially the oxidation-sensitive resveratrol. The results show that mechanical strength and PLGA content significantly affect drug-loading efficiency, penetration ability, and the physicochemical stability of RSV/NCT-CR microneedles. PLGA-MNs 3 offered the most balanced formulation among those tested, as it exhibited deformation, high drug loading, and stability. These attributes enabled reliable transdermal delivery of the PLGA-MN system for anti-wrinkle and skin rejuvenation claims.

### 3.4. Characterization of Optimized Microneedle

#### 3.4.1. Morphological Characterization

The SEM images (Figure 5f) confirmed the successful fabrication of microneedles, which are uniformly dispersed and possess smooth surfaces. PLGA-MNs 3 displayed the most uniform geometry and the fewest surface defects, while PLGA-MNs 1 showed slightly rounded tips and surface irregularities, which can be correlated to lower mechanical strength. The morphology observed is consistent with the success of the refined casting mold design and the two-step casting technique in achieving uniform microneedle production.

#### 3.4.2. Fourier Transform Infrared (FTIR) Spectroscopy

FTIR spectroscopy (Figure 4b) was used to evaluate the microneedle excipients and the drug, as well as interactions in the formulation systems. Microneedles made of PLGA exhibited an ester carbonyl signal in the region of 1750–1760 cm^−1^ and C-O-C stretching in the region of 1080–1180 cm^−1^. These peaks aligned with the drug-related peaks present in the RSV/NCT-CRs-loaded PLGA MNs, confirming the chemical compatibility of the drug encapsulation and microneedle manufacturing processes. The absence of new peaks in all formulations indicates the absence of covalent bonds and potential chemical stability.

#### 3.4.3. Differential Scanning Calorimetry (DSC)

The differential scanning calorimetry (DSC) thermographs (Figure 4c) further elucidated the physical states of RSV and NCT contained in the formulations. RSV/NCT-CRs-loaded PLGA microneedles thermographs revealed that the melting points of RSV and NCT were absent, while a broad glass transition and a thermal event of the PLGA matrix were observed around 45–60 °C, consistent with PLGA. The absence of drug-related endotherms in this system indicates effective drug encapsulation in both cerosomes and microneedles; therefore, this system is more physically stable and may improve bioavailability.

#### 3.4.4. In Vitro Drug Release Behavior

**Figure 6:**
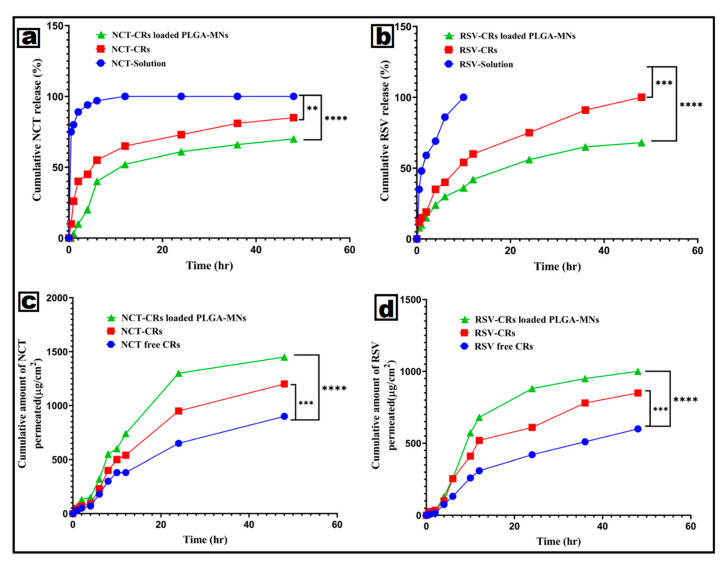
In vitro drug release and ex vivo permeation profiles of RSV and NCT formulations. (**a**) Cumulative in vitro release (%) of nicotinamide (NCT) from NCT solution, NCT-loaded cerosomes (NCT-CRs), and NCT-CRs loaded PLGA microneedles (PLGA-MNs), (**b**) Cumulative in vitro release (%) of resveratrol (RSV) from RSV solution, RSV-CRs, and RSV-CRs loaded PLGA-MNs under identical dissolution conditions, (**c**) Ex vivo cumulative permeated amount of NCT from NCT-CRs loaded PLGA-MNs, NCT-CRs, and free NCT-CRs, and (**d**) Ex vivo cumulative permeated amount of RSV from RSV-CRs loaded PLGA-MNs, RSV-CRs, and free RSV-CRs under the same experimental conditions. Data are presented as mean ± SD (n = 3). Statistical significance was determined using one-way ANOVA followed by post-hoc analysis, where ** *p* < 0.01, *** *p* < 0.001 and **** *p* < 0.0001.

The in vitro release profiles of nicotinamide (NCT) and resveratrol are shown in Figure 6a and Figure 6b, respectively. The release profiles of the solutions of the unencapsulated drugs showed a burst release in the first few hours of the experiment, with NCT reaching 95–100% release in 6–8 h, and RSV reaching 90–100% release in 8–10 h, which suggests the absence of any diffusional barriers. The release durations for cerosome formulations were significantly extended. The release of NCT-CRs was 55% at 6 h, 70% at 24 h, and 85% at 48 h. NCT-CRs combined with PLGA-MNs, however, showed a more consistent profile: 40% at 6 h, 60% at 24 h, and 70% at 48 h. At 24 h, RSV-CRs released about 80%, and at 48 h, they released 95–100%. In contrast, using PLGA-MNs with RSV-CRs resulted in slower release, achieving only 65–70% at 48 h. The cerosomal lipid bilayer and PLGA form a dual diffusion barrier, reducing the initial burst release and prolonging PLGA-MN release. The slower release of RSV compared to NCT is consistent with its greater lipophilicity and stronger interactions with lipidic and polymeric domains. NCT and RSV released from CRs and PLGA-MNs were best described by the Higuchi and Korsmeyer-Peppas models, with R^2^ values greater than 0.97, while first-order kinetics best described the release from solution formulations (R^2^ approximately 0.94–0.95). The Higuchi model R^2^ was 0.98 for NCT-CRs in PLGA-MNs. For the Korsmeyer and Peppas model, the R^2^ was 0.99 with a diffusion exponent of n = 0.41, which is characteristic of Fickian diffusion (n ≤ 0.5). The PLGA-MNs with RSV-CRs released profile was diffusion-controlled (Higuchi R^2^ = 0.97, Korsmeyer-Peppas R^2^ = 0.99, n = 0.43) as shown in Table 7. The Korsmeyer-Peppas model best described the release mechanism, as evidenced by higher R^2^ values and better characterization.

#### 3.4.5. Ex Vivo Permeation Characteristics

The chicken pouch membrane is significantly crossed by PLGA MNs, as shown by their ex vivo drug transport studies (Figure 6c,d), despite their slow in vitro release. In the 48-h study, cumulative NCT penetration from PLGA MNs was approximately 1450 µg/cm^2^, about 1200 µg/cm^2^ for NCT-CRs, and about 900 µg/cm^2^ for the free CRs. The total amount of RSV permeated from RSV/NCT-CRs-PLGA MNs (~1000 μg/cm^2^) was significantly higher than that from free RSV solution (~600 μg/cm^2^) and RSV/NCT-CRs (~850 μg/cm^2^), confirming the superior permeation-enhancing effect of the microneedle system. Microchannel formation from microneedles, which is an effective means of overcoming the primary diffusion barrier, is the most likely cause of the improved RSV and is, in fact, much more penetrative than RSV due to the high-water solubility and low molecular weight of NCT. Microneedle-assisted delivery (MN-CRs) is an effective means of distinguishing release rate from permeation efficiency, as it provides sustained local availability and improved tissue penetration through the diffusion barrier. NCT consistently outperformed RSV in terms of penetration due to its better water solubility and lower molecular weight. Microneedle-assisted delivery either decouples the release rate from permeation efficiency or maintains the release rate to promote sustained local presence and improve permeation. The release and permeation data show that RSV/NCT-CRs within the PLGA-MN matrix achieved sustained drug release (65–70% over 48 h) and ex vivo penetration enhancement (up to 1.6 times) compared to devices without microneedles. Among the applied kinetic models, the Korsmeyer-Peppas model best described the data, suggesting that Fickian diffusion is the primary mechanism of release. This approach is particularly well-suited for the treatment of wrinkles as it is imperative on having the drug remain within the skin for an extended period of time, which to attain a sustained therapeutic outcome is critical.

### 3.5. In Vivo Study

Photoaging is a complex degenerative process brought on by prolonged exposure to UVA light. Increased oxidative stress, the start of inflammatory reactions, interference with skin regeneration signaling pathways, and a slow deterioration of the extracellular matrix are characteristics of this process. The study assessed in vivo research on skin photodamage and aging. In particular, the study evaluated the skin reparative properties of the transdermal RSV/NCT cerosome combination delivered via PLGA microneedles in a mouse model of UVA-induced skin aging. The study demonstrates the effectiveness of microneedle-assisted transdermal RSV/NCT delivery combined with CRM/PLGA MNs in achieving anti-wrinkle results that exceed those of other topical formulations.

#### 3.5.1. Macroscopic Skin Appearance

**Figure 7:**
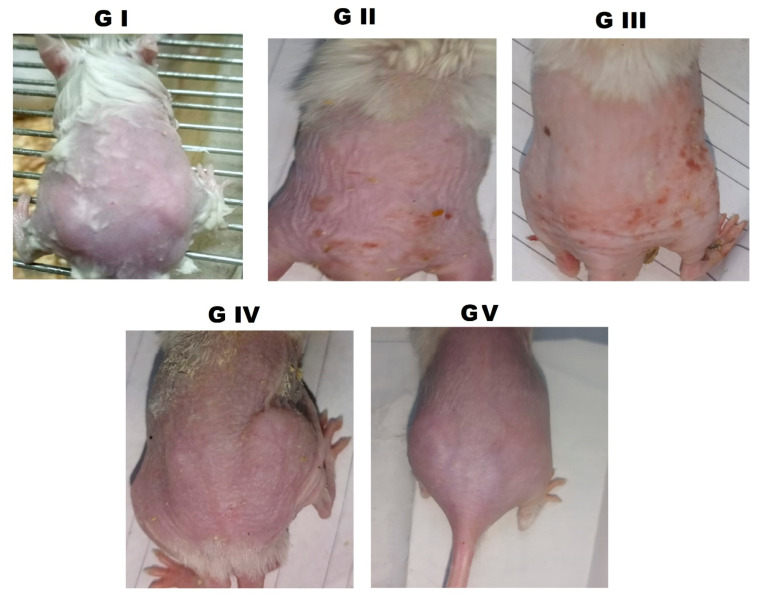
Macroscopic appearance of dorsal skin in UVA-induced wrinkle mouse model following different treatments; GI: Healthy control mice without UVA exposure, GII: UVA-irradiated untreated mice (positive control), GIII: mice treated with RSV/NCT gel (2% *w*/*w* HPMC), GIV: treated with RSV/NCT-CRs gel (2% *w*/*w* HPMC), and GV: treated with RSV/NCT-CRs-loaded PLGA microneedle (MN) patch.

The skin features of the treated groups reflect biochemical and molecular improvements. Creeping and wrinkled mice (GII) without treatment showed a coarse skin, pronounced wrinkles, and erythema, which signify pronounced skin photoaging (Figure 7). GIII and GIV showed partial vision enhancement, yet only the RSV/NCT-CRs-PLGA MNs group (GV) showed skin morphology close to normal, with skin surfaces much smoother and fewer wrinkles. The advancement demonstrated from RSV–NCT gel (GIII) to RSV–NCT-CRs gel (GIV), and then to RSV–NCT-CRs-PLGA MNs (GV), shows that both the nanocarrier design and microneedle-assisted delivery have a positive impact on the formulation’s therapeutic efficacy. Such a tiered ranking system suggests that cerosomal encapsulation increases retention and stability in the skin. Microneedles can bypass the stratum corneum and deliver the dual-drug system to the skin layers most affected by UV radiation. Throughout the treatment period, animals receiving RSV–NCT-CRs-PLGA microneedles did not exhibit visible signs of severe skin irritation, ulceration, or behavioral distress at the application site, suggesting acceptable local tolerability under the dosing conditions used. These results show that substantial improvements in skin aesthetics also indicate that molecular and cellular repair processes are at work.

#### 3.5.2. Modulation of Inflammatory Cytokines Assessed by ELISA

**Figure 8:**
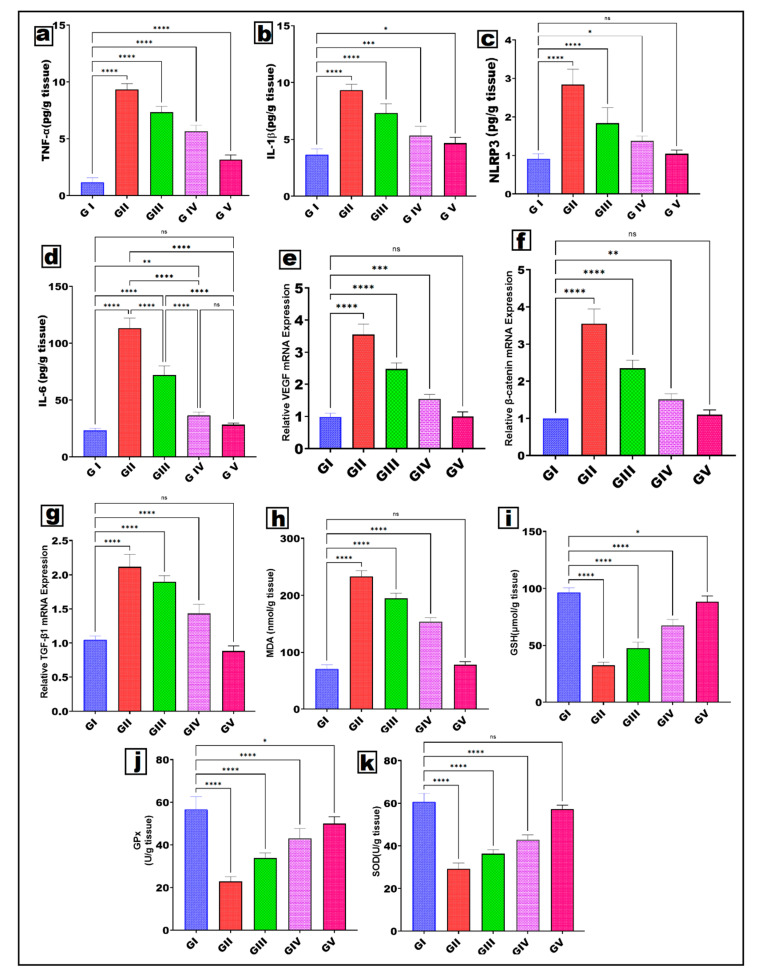
Effects of different treatments on inflammatory biomarkers measured by ELISA: (**a**) TNF-α, (**b**) IL-1β, (**c**) NLRP3, and (**d**) IL-6. qRT-PCR analysis demonstrating the relative transcription levels of VEGF (**e**), β-actin (**f**), and TGF-β1 (**g**). Oxidative stress analysis via MDA (**h**), GSH (**i**), GPx (**j**), and SOD (**k**). Data are expressed as mean ± SD (n = 10). * *p* < 0.05, ** *p* < 0.01, *** *p* < 0.001, **** *p* < 0.0001, and ns (not significant) versus the control group. Abbreviations: GI: Healthy control mice without UVA exposure, GII: UVA-irradiated untreated mice (positive control), GIII: mice treated with RSV/NCT gel (2% *w*/*w* HPMC), GIV: treated with RSV/NCT-CRs gel (2% *w*/*w* HPMC), and GV: treated with RSV/NCT-CRs-loaded PLGA microneedle (MN) patch. Data were expressed as Mean ± SD, n = 3.

Figure 8a shows that the untreated UVA-exposed group (GII) had significantly higher tumor necrosis factor-α (TNF-α) levels than the healthy control group (GI). This verifies that inflammatory photoaging was effectively generated. TNF-α is an essential upstream cytokine that activates nuclear factor-κB (NF-κB) signaling and stimulates matrix metalloproteinase (MMP) synthesis, leading to collagen degradation. The treatment with RSV/NCT gel (GIII) led to a notable decrease in TNF-α levels; however, these levels remained higher than those in healthy skin. The RSV/NCT-CRs gel group (GIV) showed a notably greater reduction, suggesting improved drug stabilization and extended release, thereby enhancing its effectiveness in combating inflammation. The group with RSV/NCT-CRs-loaded PLGA microneedles (GV) demonstrated the greatest reduction in TNF-α, approaching baseline levels. This suggests that microneedle delivery is more effective at suppressing inflammatory signals triggered by UVA exposure. Interleukin-1β (IL-1β), an essential mediator of inflammation triggered by the inflammasome, exhibited a comparable pattern (Figure 8b). Exposure to UVA radiation significantly elevated IL-1β levels in GII, indicating activation of the NLRP3 inflammasome pathway. The RSV/NCT gel treatment (GIII) led to a notable decrease in IL-1β expression, though it was only partial. Encapsulating RSV/NCT in cerosomes (GIV) further reduced IL-1β levels. The group undergoing microneedle therapy (GV) showed the most notable decrease. This data shows that the successful distribution of RSV/NCT on the skin is crucial for effectively inhibiting cytokine maturation mediated by the inflammasome. In alignment with the findings related to IL-1β, the expression of NLRP3 protein (Figure 8c) was notably increased in the UVA-irradiated untreated group (GII) when compared to GI, thereby indicating the activation of the inflammasome in photoaged skin. All treated groups showed a decrease in NLRP3, with RSV/NCT-CRs-PLGA MNs (GV) showing the most notable reduction. The significant decrease in NLRP3 in GV suggests a successful suppression of inflammasome assembly, thereby mitigating the ensuing inflammatory response and tissue injury. Interleukin-6 (IL-6), a versatile cytokine linked to chronic inflammation and collagen breakdown, showed a significant rise in GII (Figure 8d). Excessive IL-6 accelerates dermal matrix degradation and diminishes skin elasticity. The use of RSV/NCT gel and RSV/NCT-CRs gel significantly reduced IL-6 levels, with microneedle delivery being most effective in restoring levels to normalcy. The superior IL-6 suppression by GV supports the notion that microneedle technology is more effective at combating inflammation. The ELISA results demonstrate that RSV/NCT formulations efficiently reduce UVA-induced inflammatory responses, while their efficacy differs by formulation. The levels of TNF-α, IL-1β, NLRP3, and IL-6 consistently decreased after integrating RSV/NCT-CRs into PLGA microneedles. An improved anti-inflammatory effect is achieved by combining RSV’s potent antioxidant and anti-inflammatory properties with NCT’s ability to restore metabolism, stabilize cerosomes, and facilitate transdermal delivery via microneedles. The advancement demonstrated from RSV–NCT gel (GIII) to RSV–NCT-CRs gel (GIV), and then to RSV–NCT-CRs-PLGA MNs (GV), shows that both the nanocarrier design and microneedle-assisted delivery have a positive impact on the formulation’s therapeutic efficacy. Such a tiered ranking system suggests that cerosomal encapsulation increases retention and stability in the skin. Microneedles can bypass the stratum corneum and deliver the dual-drug system to the skin layers most affected by UV radiation [96,97]. The notable reduction in inflammatory mediators provides mechanistic support for the observed improvements in skin architecture and reduced wrinkles, thereby affirming the therapeutic efficacy of the developed microneedle technology for photoaging treatment. Restore regenerative and extracellular matrix-related signaling pathways.

#### 3.5.3. qRT-PCR Analysis

Photoaging resulting from prolonged UVA exposure is associated with inflammation and oxidative stress, as well as a notable suppression of critical molecular pathways that promote skin regeneration and extracellular matrix (ECM) remodeling. This study employed qRT-PCR to quantify VEGF, β-catenin, and TGF-β1 levels and assess the impact of various RSV/NCT administration routes on the body’s skin-healing capacity. UVA radiation significantly accelerates skin photoaging by inducing oxidative stress, inflammatory responses, dysregulation of angiogenesis, and disruption of intracellular signaling pathways crucial for epidermal and dermal homeostasis (Figure 8e,f). Relative VEGF and β-catenin mRNA expression, along with other molecules, primarily regulate the formation of UVA-induced wrinkles, the degradation of the dermal matrix, and abnormal skin remodeling. Mice in GpII showed a marked increase in VEGF and beta-catenin expression. The increase was statistically significant (****, *p* < 0.0001). UVA radiation exposes skin cells to Reactive Oxygen Species (ROS), which activate NF-kB and AP-1. This leads to overexpression of VEGF and results in pathological angiogenesis (a consequence of photoaging). UVA radiation activates the Wnt/beta-catenin pathway, which leads to excessive proliferation of keratinocytes, which in turn leads to the thickening of the epidermis and loss of the dermal-epidermal junction. All these factors contribute to the development of wrinkles and to structural damage to the skin. This is confirmation that GpII is a positive/valid photoaging control. GpIII (the 2% *w*/*w* HPMC (hydroxypropyl methylcellulose) gel with RSV/NCT) was the only treatment to produce a statistically significant (****, *p* < 0.0001) reduction in expression of VEGF and beta-catenin compared to GpII. This is attributable to the known biological effects of components of RSV/NCT gel. RSV is a potent polyphenolic antioxidant that scavenges ROS, inhibits MAPK signaling, and reduces UV-induced production of inflammatory mediators, thereby lowering VEGF overexpression. NCT is a precursor of NAD+, which supports cellular processes that improve energy metabolism, DNA repair, and inflammation, which help control β-catenin signaling. Although improvements in the molecules were observed, levels of both VEGF and β-catenin in GpIII remained higher than in healthy controls. The low rate of normalization is probably because most topical gels have limited skin penetration, are rapidly cleared from the skin, and poorly deliver the drug to target cells. Greater clinical efficacy of RSV/NCT-Loaded CRs (GpIV). The combination of GpIV and RSV/NCT formulated in a cerosomes nanovesicle (CR)-HPMC gel resulted in a marked decline of β-catenin and VEGF levels that was statistically significant (****, *p* < 0.0001). Cerosomes are lipid nanocarrier systems that mimic biological membranes. They improve drug stability, increase skin retention time, and facilitate cellular uptake. The increased molecular suppression seen in GpIV compared to GpIII suggests that CRs operate efficiently. Despite significant improvements in therapeutic effects with CR-based administration, the expression levels of VEGF and β-catenin remained elevated above normal levels. This indicates that topical nanoformulations may continue to face distribution challenges, as the stratum corneum remains intact. RSV/NCT-CRs-PLGA Microneedles Show Superior Efficacy (GpV). The most dramatic therapeutic effect was observed in GpV, with UVA-irradiated mice treated with RSV/NCT-CRs-loaded PLGA microneedle (MN) patches. This group showed nearly complete normalization of VEGF and β-catenin mRNA expression, with no statistically significant difference (ns) compared with healthy control mice (GpI) and a highly significant reduction (***, *p* < 0.0001) compared with GpII and GpIII. This delivery system’s greater efficacy is mechanistically explained by PLGA microneedles’ ability to bypass the stratum corneum barrier, directly deliver RSV/NCT-CRs into viable epidermal and upper dermal layers, and maintain intracellular drug concentrations by controlling PLGA degradation. Targeted transdermal administration effectively suppresses UVA-induced angiogenic signaling and modulates Wnt/β-catenin signaling, restoring epidermal signaling balance. The observed molecular normalization is closely correlated with enhanced skin structure and the prevention of photoaging-related pathological changes. (Figure 8g), show TGF-β1 mRNA expression, TGF-β1 regulates dermal extracellular matrix homeostasis, collagen turnover, and tissue remodeling. These processes are dysregulated by photoaging and UV-induced wrinkling in skin [98,99]. In this study, healthy control mice (GpI) had normal levels of TGF-β1 mRNA, reflecting the natural cellular signaling that produces and breaks down collagen. In the opposite case, untreated UVA-irradiated wrinkled mice (GpII) showed higher TGF-β1 expression than did normal mice. This shows that there are exposure-induced, chronic, photodamage profibrotic changes and remodeling pathways actively. Previous studies show that chronic exposure to UVA increases TGF-β1 signaling, leading to disorganized collagen, reactivation of the dermal matrix, and, eventually, wrinkled skin. Compared with GpII, GpIII showed lower TGF-β1 mRNA levels, reflecting lower dermal penetration of RSV and NCT. Even though both drugs are antioxidants and anti-inflammatories that affect TGF-β, their lack of skin penetration affected therapeutic outcomes. Although they showed some remodeling improvements, the overexpression of TGF-β1 suggests they did not completely restore skin structure or reduce wrinkles. This approach crosses the stratum corneum barrier, RSV/NCT-loaded carriers to the epidermis and dermis and sustains active component release longer than PLGA-based microneedles. This combination may improve the anti-oxidative and anti-inflammatory activity and enhance TGF-β/Smad signaling. Microneedle technology stimulates physiological matrix remodeling, leading to organized collagen deposition which increases skin elasticity and anti-wrinkle effects. The qRT-PCR data support that RSV/NCT-based therapies successfully reverse IMF-UVUA-inhibition of important pathways of cell growth and repair. Among the formulations, microneedles containing PLGA RSV/NCT-CRs showed the most promising molecular profile. This was the result of restoring angiogenesis via VEGF, modulating the β-catenin pathway, and normalizing ECM production driven by TGF-β1. The activity of microneedles and nanocarriers targeted to the molecular-level deficiencies of photoaging explains improvements in collagen production, skin architecture, and reduced wrinkles [96]. Mice treated with RSV/NCT-CRs gel (GpIV) showed a notable reduction in TGF-β1 expression, demonstrating the advantage of using colloidal carriers [97]. The formulation based on a colloidal carrier reduces oxidative stress and inflammation caused by UVA, in addition to improving the stability of the medication, its penetration into the skin, and its retention in the dermis. Intermediate TGF-β1 levels in GpIV, which are lower than GpII and GpIII but higher than GpI, are indicative of some restoration of collagen organization and reduction in wrinkles. This is consistent with a significant, yet incomplete, response to remodeling associated with photoaging. Among the groups, the greatest TGF-β1 mRNA normalization was observed in the RSV/NCT-CRs-PLGA microneedle patch-treated group (GpV). TGF-β1 levels in Gp. I was at baseline levels, indicating a significant reduction in the UVA-induced increase [98]. This technique surpasses the stratum corneum barrier, and RSV/NCT-loaded carriers are dispersed into the epidermis and dermis, which sustain active component release for a longer duration compared to the PLGA-based microneedles. This combination may improve efficacy of the treatments by the lowering of the oxidative and inflammatory processes and increasing the TGF-β/Smad signaling. Microneedle technology increases physiological matrix turnover which organizes collagen remodeling and enhances skin elasticity for better laxity and suppleness which reinforces the anti-wrinkle effects. The qRT-PCR analysis show that therapies based on RSV/NCT alleviate the most UVA-induced blockage of the pathways pivotal to the cell’s growth and repair. Amongst all the formulations, the most promising molecular profile was the PLGA microneedles containing RSV/NCT-CRs. This was shown by the reinstatement of VEGF-mediated angiogenesis, the regulation of β-catenin signaling, and the restoration of the TGF-β1-induced extracellular matrix. Microneedle nanocarrier technology efficiently addresses the molecular deficits of photoaging, improves collagen formation and epidermal structure, and reduces wrinkles.

#### 3.5.4. Oxidative Stress Modulation and Antioxidant Defense Recovery

Figure 8h–k shows how oxidative stress and antioxidant capacity were assessed using malondialdehyde (MDA), reduced glutathione (GSH), glutathione peroxidase (GPx), and superoxide dismutase (SOD) in homogenates of skin tissues.

Figure 8 shows that, compared with the healthy control group (GI), the untreated UVA-irradiated group (GII) has significantly higher levels of MDA. This confirms that photoexposure has caused considerable lipid peroxidation and membrane damage. This increase results from ROS-mediated oxidation of the polyunsaturated fatty acids in cellular membranes, a characteristic of photoaged skin. Application of the RSV/NCT gel (GIII) reduced MDA levels, indicating that some oxidative damage has been lessened. The RSV/NCT-CRs gel group (GIV) showed a greater difference, suggesting that the antioxidant’s efficacy was enhanced because the drugs’ stability and sustained release were improved. Among all the treated groups, the RSV/NCT-CRs-PLGA MNs (GV) group had the lowest MDA levels, which were nearly identical to those in healthy skin. This indicates that microneedle delivery is more effective at reducing lipid peroxidation. The significant decrease in GII’s GSH levels (Figure 8i) after exposure to UVA showed the deficiency of the cellular antioxidant defense mechanisms. Glutathione (GSH) is a vital intracellular antioxidant that neutralizes free radicals and is also a substrate for antioxidants. The administration of RSV/NCT formulations significantly replenished GSH levels, depending on the formulation used. The RSV/NCT gel and RSV/NCT-CRs gel partially restored GSH levels, but the greatest enhancement was in the GV (microneedles) group. This indicates successful restoration of redox balance, which could also be due to the enhancement of NAD^+^-dependent (and redox) metabolic pathways by nicotinamide and to the radical-scavenging effect of red wine polyphenols (resveratrol). Similarly, in the untreated UVA-irradiated group, the critical antioxidant enzyme activities of GPx and SOD were significantly suppressed, indicating exhaustion of the enzymatic defense mechanisms due to increased ROS (reactive oxygen species) (Figure 8j,k). GPx is vital for the breakdown of hydrogen peroxide and lipid hydroperoxides, while SOD catalyzes the conversion of superoxide radicals to less reactive species. The RSV/NCT gel treatment partially restored the GPx and SOD activity, and the RSV/NCT-CRs gel treatment had a greater effect. RSV/NCT-CRs-PLGA MNs had the most GPx and SOD activity, meaning there was a near- complete restoration of enzymatic antioxidant capacity. There are multiple interconnected reasons for the enhanced antioxidant effect in the microneedle group. To begin with, resveratrol directly removes reactive oxygen species (ROS) and increases the activity of existing antioxidant enzymes through redox-sensitive signaling pathways. Secondly, nicotinamide improves energy utilization at the cellular level and increases antioxidant capacity by elevating cellular NAD^+^. Third, cerosomal encapsulation protects RSV and NCT from degradation, and PLGA microneedles bypass the stratum corneum to target the active ingredients to the most oxidatively damaged layers of the skin. RSV/NCT-CRs-PLGA MNs, as per the data, reduce UVA-induced oxidative stress by restoring antioxidant (both enzymatic and non-enzymatic) defenses and inhibiting lipid peroxidation. The normalization of markers of oxidative stress translates to the molecular basis of the claimed improvement in the appearance of wrinkles and skin texture, the activation of regenerative processes, and a reduction in inflammation. The normalization of oxidative stress and inflammatory biomarkers observed with RSV/NCT-CRs-PLGA MNs agrees with previous reports in which antioxidant-loaded nanocarriers or microneedle systems attenuated UVA-induced increases in MDA and pro-inflammatory cytokines while restoring dermal collagen architecture. The evidence also supports the microneedle-based delivery of nanocarriers as a valuable means of alleviating the oxidative effects of UV radiation on the skin.

### 3.6. Histopathology Study

**Figure 9:**
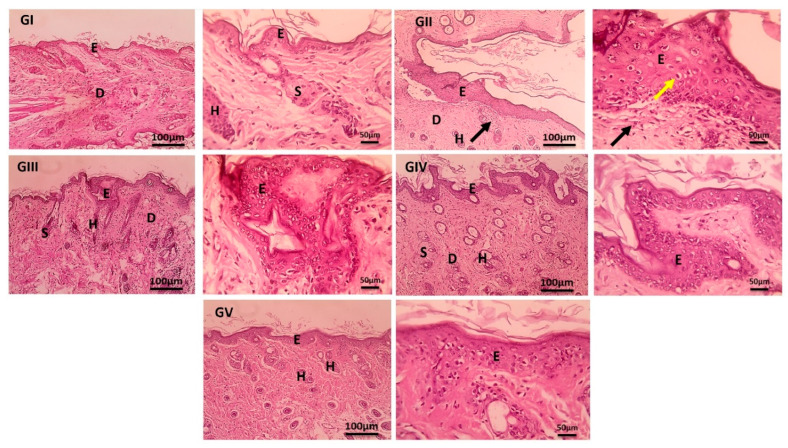
Hematoxylin and eosin (H&E)–stained sections of dorsal skin from all experimental groups. For each group, the left image (×100) shows overall epidermal and dermal architecture, while the right image (×400) highlights epidermal thickness and cellular detail. Scale bars: 100 μm (left panels) and 50 μm (right panels).Microscopic pictures of HE-stained skin sections from control normal group (GI) showing normal epidermis (E) and dermis (D) containing collagen, hair follicles (H), sebaceous glands (S). Skin sections from untreated GII showing markedly thickened epidermis (E) due to hyperkeratosis&acanthosis with ballooning degeneration (yellow arrows) besides sub-epidermal inflammatory cells infiltrates (thin black arrows). Skin sections from treated GIII showing focally thickened epidermis (E) due to hyperkeratosis&acanthosis with normal hair follicles (H), sebaceous glands (S) in dermis (D). Skin sections from treated GIV showing decreased epidermal thickening (E) with normal hair follicles (H), sebaceous glands (S) in dermis (D). Skin sections from treated GV showing markedly decreased epidermal thickening (E) with normal hair follicles (H), sebaceous glands (S) in dermis (D)(Low magnification ×100 bar 100 and high magnification ×400 bar 50). **Abbreviations**: GI: Healthy control mice without UVA exposure, GII: UVA-irradiated untreated mice (positive control), GIII: mice treated with RSV/NCT gel (2% *w*/*w* HPMC), GIV: treated with RSV/NCT-CRs gel (2% *w*/*w* HPMC), and GV: treated with RSV/NCT-CRs-loaded PLGA microneedle (MN) patch. Data were expressed as Mean ± SD, n = 3.

Histopathological investigation of skin sections tinted with H&E (Figure 9) demonstrates that skin samples from the healthy control group (GI) showed a proper, characteristic architecture with a thin and organized epidermis (E) and an intact dermis (D) with the deep layers showing abundant and uniformly arranged collagen fibers. The hair follicles (H) and sebaceous glands (S) were normally distributed, confirming the homeostasis and structural integrity of the skin. The results elucidate the molecular basis of healthy skin. In contrast, skin slices from the untreated UVA-irradiated group (GII) exhibited severe histological abnormalities characteristic of photoaging. The findings showed major thickening of the outer skin layer due to keratin production and increased cell mitoses, suggesting a response to prolonged UVA exposure. Ballooning keratinocyte degeneration, as indicated with yellow arrows, demonstrates cell damage and swelling due to cadherin alterations and oxidative stress. Increased H&E skin sectioning demonstrates the structural effects of UVA and the therapeutic effects of RSV/NCT. From a morphological perspective, the experimental groups differ significantly in their demonstration of photoaging and its reversal. Skin samples from this group showed localized epidermal thinning, which suggests a decrease, but not a total elimination, of the hyper-proliferative response. The dermal layers that preserved the hair follicles and sebaceous glands show some degree of structural restoration [100,101,102]. The remaining skin imperfections indicate the shallow absorption and limited depth of action of the dermal layers with the use of the conventional topical gel. The RSV/NCT-CR gel-treated group (GIV) presented with additional improvements. The histological sections showed that the epidermis was thinner and more organized than that of GIII. The dermis contained healthy hair follicles and sebaceous glands, signifying good tissue repair. This improvement can be attributed to the more sustained release and the enhanced stability of RSV/NCT, when entrapped in cerosomes, which permits the prolonged interaction with skin tissues.

### 3.7. Masson’s Trichrome MT Staining

**Figure 10:**
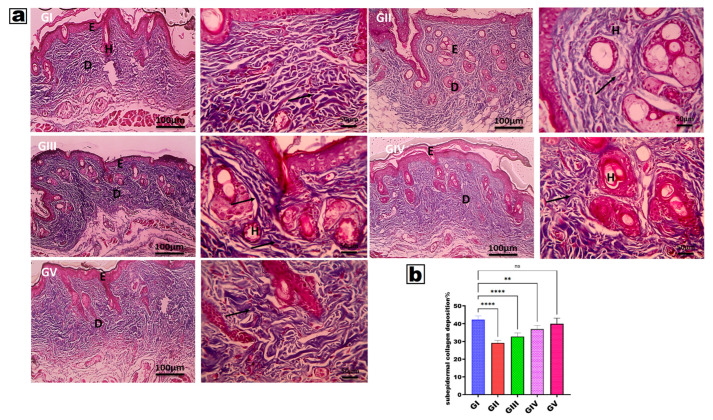
(**a**) Masson’s trichrome–stained sections of dorsal skin illustrating collagen distribution and organization showing normal epidermis (E) and dermis (D) containing normal subepidermal collagen density in G1 control normal group, increased epidermis thickness, increased number of hair follicles (H) with decreased subepidermal collagen deposition in dermis (D) of untreated GII, subepidermal collagen deposition in dermis (D) (thin black arrows) increased gradually in treatment groups GIII, GIV, GV associated with decreased epidermal thickness, number of hair follicles; (**b**) Subepidermal collagen deposition (Low magnification ×100 bar 100 and high magnification ×400 bar 50). ** *p* < 0.01, **** *p* < 0.0001, and ns (not significant) versus the control group. Thin black arrows indicate a progressive increase in subepidermal collagen deposition.

Masson’s Trichrome (MT) staining was employed to analyze dermal collagen structure and subepidermal collagen accumulation in the experimental groups (Figure 10a). In the control normal group (GI), MT-stained skin sections showed a well-structured epidermis (E) and dermis (D) with robust, uniformly distributed subepidermal collagen fibers, indicating preserved dermal integrity and a normal extracellular matrix configuration. Group II (GII) exhibited the most considerable histological change, which consisted of a thickening of the epidermis and a greater size of hair follicles (H) with a reduction in collagen deposition subepidermally. The collagen disarray and depletion reflect UV-induced damage to the dermal collagen network, most likely due to accelerated collagen catabolism and reduced fibroblast function because of oxidative and inflammatory stress [101,103]. The results suggest that, relative to the untreated model, the untreated model led to alterations consistent with wrinkles and photoaging. GIII, GIV, and GV all exhibited greater collagen deposition than GII. GIII, GIV, and GV showed greater collagen deposition than GII. GV had skin collagen structure and density greater than all the other groups; this was like the normal control, suggesting that GV had the greatest ability to restore the integrity of the dermal extracellular matrix [104,105]. The quantitative analysis of subepidermal collagen deposition (Figure 10b) corroborated the histological observations, indicating a significant reduction in collagen levels in GII relative to GI, succeeded by a gradual increase in the treated groups. Reduced inflammatory damage and increased fibroblast activity may have facilitated the maintenance of collagen and/or the production of new collagen. MT staining demonstrates that the treatments reduced UV-induced collagen degradation and facilitated dermal remodeling, consequently improving skin structure and decreasing wrinkle formation.

## 4. Conclusions

This study successfully developed an enhanced transdermal delivery system utilizing resveratrol and nicotinamide-loaded cerosomes within PLGA-based microneedles, which were rigorously evaluated for anti-aging and anti-wrinkle treatment. This study addressed the primary issues associated with conventional topical delivery of resveratrol and nicotinamide, including inadequate skin penetration, insufficient retention, and poor dermal absorption. The nanovesicular system, comprising phosphatidylcholine, ceramides, and poloxamer surfactants, exhibited favorable physicochemical properties, efficient drug encapsulation, and controlled drug release over time. The incorporation of customized cerosomes into a biodegradable microneedle array system is strong, yet it remains a minimally invasive delivery system that can breach the stratum corneum and improve the transdermal transport of therapeutic agents. Integrated cerosome-microneedle technology, compared to traditional formulations, enhanced skin permeation and improved the delivery of pharmaceuticals more accurately. The platform enhanced skin structure, epidermal and dermal architecture, collagen organization, and indicators of inflammation and oxidative stress in a UVA-induced skin-wrinkle model, demonstrating its therapeutic potential. Genes involved in angiogenesis and extracellular matrix remodeling are upregulated, supporting the system’s role in skin regeneration and in reducing photoaging. This study demonstrates that cerosome-loaded microneedle systems for transdermal delivery of natural anti-aging agents are effective, safe, and aligned with patient requirements. The findings support the use of this platform for sophisticated dermatological and cosmeceutical therapies, as well as for future translational and clinical research.

## Figures and Tables

**Table 1:** D-Optimal factorial design utilized for optimizing RSV-CR formulations.

**Factors (Independent Variables)**	**Design Levels**		
	Low (−1)	Medium (0)	High (+1)
**X1: PC amount (mg)**	50	75	100
**X2: Ceramide type**	III	IIIB	IV
**X3: SAA type**	Pluronic F127	Pluronic 188	Pluronic L121
**Responses (Dependent variables)**	Goal		
**Y1: EE(%)**	Maximize		
**Y2: PS (nm)**	Minimize		
**Y3: PDI (nm)**	Minimize		
**Y4: ZP (mV)**	Maximize		

**Abbreviations**: PC, Phosphatidylcholine; SAA, Surface active agent; PS, Particle size; PDI, polydispersity index; ZP, zeta potential, and EE, entrapment efficiency.

**Table 2:** Composition of RSV/NCT-loaded Cerosomes with their measured responses (n = 3 ± SD).

Run	Factors			Responses				
	A: PC Amount (mg)	B: Ceramide Type	C: SAA Type	EE RSV%	EE NCT %	PS nm	PDI nm	ZP mV
1	100	IIIB	Pluronic L121	91 ± 0.58	76 ± 0.43	180 ± 0.88	0.21 ± 0.02	−26 ± 0.23
2	100	III	Pluronic L121	87 ± 0.24	63 ± 0.67	190 ± 0.53	0.23 ± 0.04	−24 ± 0.18
3	50	IV	Pluronic 188	54 ± 0.86	56 ± 0.66	350 ± 0.85	0.33 ± 0.03	−19 ± 0.48
4	75	III	Pluronic 188	61 ± 0.83	53 ± 0.45	300 ± 0.47	0.29 ± 0.05	−21 ± 0.25
5	75	IV	Pluronic F127	70 ± 0.61	58 ± 0.72	220 ± 0.65	0.22 ± 0.04	−22 ± 0.54
6	50	III	Pluronic 188	50 ± 0.88	53 ± 0.34	310 ± 0.36	0.35 ± 0.03	−18 ± 0.61
7	100	IV	Pluronic F127	70 ± 0.85	59 ± 0.52	210 ± 0.67	0.2 ± 0.05	−21 ± 0.43
8	100	IIIB	Pluronic F127	76 ± 0.37	64 ± 0.54	240 ± 0.45	0.22 ± 0.02	−20 ± 0.51
9	100	III	Pluronic 188	50 ± 0.74	44 ± 0.65	290 ± 0.65	0.27 ± 0.04	−19 ± 0.43
10	50	IIIB	Pluronic F127	60 ± 0.34	52 ± 0.94	250 ± 0.23	0.3 ± 0.05	−18 ± 0.23
11	75	III	Pluronic F127	70 ± 0.94	69 ± 0.67	220 ± 0.86	0.33 ± 0.02	−19 ± 0.42
12	75	III	Pluronic L121	89 ± 0.98	64 ± 0.88	215 ± 0.88	0.25 ± 0.04	−23 ± 0.32
13	75	IIIB	Pluronic F127	73 ± 0.53	52 ± 0.34	200 ± 0.81	0.25 ± 0.03	−21 ± 0.41
14	75	IIIB	Pluronic L121	88 ± 0.51	63 ± 0.76	200 ± 0.46	0.2 ± 0.01	−20 ± 0.52
15	50	IV	Pluronic F127	63 ± 0.94	53 ± 0.55	270 ± 0.66	0.28 ± 0.04	−18 ± 0.53
16	50	IV	Pluronic L121	78 ± 0.65	65 ± 0.98	240 ± 0.45	0.32 ± 0.01	−20 ± 0.61
17	75	IV	Pluronic L121	90 ± 0.34	85 ± 0.99	210 ± 0.48	0.21 ± 0.04	−23 ± 0.63
18	75	III	Pluronic F127	73 ± 0.66	61 ± 0.45	220 ± 0.65	0.22 ± 0.02	−20 ± 0.55
19	100	IV	Pluronic 188	63 ± 0.72	54 ± 0.56	300 ± 0.54	0.39 ± 0.03	−21 ± 0.61
20	100	IIIB	Pluronic 188	55 ± 0.65	57 ± 0.99	410 ± 0.66	0.41 ± 0.02	−19 ± 0.43
21	50	IIIB	Pluronic 188	48 ± 0.91	52 ± 0.67	430 ± 0.39	0.44 ± 0.03	−17 ± 0.53
OF	83	IV	Pluronic L121	91 ± 0.56	85 ± 0.56	195 ± 0.78	0.23 ± 0.01	−22 ± 0.45

**Abbreviations:** RSV, resveratrol; NCT, Nicotinamide; SAA, Surface active agent; PC, Phosphatidylcholine; PS, Particle size; PDI, polydispersity index; ZP, zeta potential; EE, entrapment efficiency; and OF, optimized formula.

**Table 3:** Fabrication of PLGA-MNs loading freeze-dried Resveratrol/Nicotinamide-loaded Cerosomes (RSV/NCT-CRs).

Formulations	PLGA (*w*/*w*)	PVA (*w*/*v*)	PVP k90 (*w*/*v*)
PLGA-MNs 1	2.5	30	5
PLGA-MNs 2	5	20	10
PLGA-MNs 3	10	10	20

**Table 4:** The primer sequences used for amplification of the mouse GAPDH, TGF-β1, VEGF, and β-Catenin genes.

Primer	Sequence	NCBI Reference Sequence	Amplification Size	Annealing Temperature
β-Catenin	F: 5′-GTTCGCCTTCATTATGGACTGCC-3′ R: 5′-ATAGCACCCTGTTCCCGCAAAG-3′	NM_007614.3	146	60 °C
VEGF	F: 5′-CACGACAGAAGGAGAGCAGAAG-3′ R: 5′-CTCAATCGGACGGCAGTAGC-3′	NM_001025250.3	82	60 °C
TGF-β1	F: 5′-ACTGGAGTTGTACGGCAGTG-3′ R: 5′-GGGGCTGATCCCGTTGATTT-3′	NM_011577.2	123	60 °C
GAPDH	F: 5′-ATGGTGAAGGTCGGTGTGAAC-3′ R: 5′-TTGATGTTAGTGGGGTCTCGC-3′	NM_008084.3	251	60 °C

**Note**: VEGF: vascular endothelial growth factor; GAPDH: Glyceraldehyde 3-phosphate dehydrogenase; TGF-β1: transforming growth factor, beta 1; F: forward; R: reverse.

**Table 5:** Output data of the D-optimal design analysis of RSV-CRs.

Responses	Y1: EE RSV%	Y2: EE NCT%	Y3: PS	Y4: PDI	Y5: ZP
Minimum	48	44	180	0.2	−26
Maximum	91	85	430	0.44	−17
Model	Linear	2F1	2F1	Linear	Linear
*F*-value	82.71	22.46	151.53	0.019	12.69
*p*-value	<0.0001	0.0009	0.011	0.001	0.0020
R^2^	0.9252	0.9200	0.9825	0.6420	0.7053
Adjusted R^2^	0.9003	0.7716	0.9500	0.5226	0.6071
Predicted R^2^	0.8526	0.2107	0.9005	0.2783	0.4406
Adequate Precision	17.1609	10.3557	19.3374	7.3049	8.9214
Significant factors	A, C	C	A, B, C	A, C	A, C

**Table 6:** The stability study of the optimized RSV/NCT-CRs formulation under both storage conditions.

Storage Time	Refrigerated Temperature (4 ± 1 °C)					Ambient Temperature				
	EE RSV (%)	EE NCT (%)	PS (nm)	PDI (nm)	ZP (mV)	EE RSV (%)	EE NCT (%)	PS (nm)	PDI (nm)	ZP (mV)
After 24 h	91 ± 0.56	85 ± 0.56	195 ± 0.78	0.23 ± 0.01	−22 ± 0.45	90 ± 0.34	84 ± 0.11	195 ± 0.01	0.24 ± 0.02	−21 ± 0.12
3 months	88 ± 0.89	81 ± 0.67	199 ± 0.45	0.26 ± 0.08	−20 ± 0.45	86 ± 0.78	79 ± 0.43	204 ± 0.76	0.27 ± 0.09	−28 ± 0.12
6 months	85 ± 0.34	79 ± 0.39	201 ± 0.65	0.31 ± 0.05	−21 ± 0.45	83 ± 0.88	76 ± 0.91	209 ± 0.88	0.31 ± 0.06	−34 ± 0.12

Data are displayed as mean ± SD from three independent tests (n = 3). **Abbreviations**: RSV, resveratrol; NCT, Nicotinamide; PS, Particle size; PDI, polydispersity index; ZP, zeta potential; EE, entrapment efficiency.

**Table 7:** Kinetic modeling of RSV and NCT in vitro release profiles.

Formulation	Zero-Order R^2^	First-Order R^2^	Higuchi R^2^	Korsmeyer–Peppas R^2^	n Value	Release Mechanism
NCT-Solution	0.78	0.95	0.82	0.89	0.82	Concentration-dependent
NCT-CRs	0.90	0.88	0.96	0.97	0.46	Fickian diffusion
NCT-CRs-PLGA-MNs	0.92	0.85	0.98	0.99	0.41	Fickian diffusion
RSV-Solution	0.74	0.94	0.80	0.86	0.88	Concentration-dependent
RSV-CRs	0.88	0.86	0.95	0.96	0.48	Fickian diffusion
RSV-CRs-PLGA-MNs	0.91	0.83	0.97	0.99	0.43	Fickian diffusion

## Data Availability

The data presented in this study are available on request from the corresponding authors.

## Abbreviations

Abbreviation	Full Term
RSV	Resveratrol
NCT	Nicotinamide
MNs	Microneedles
PLGA	Poly(lactic-co-glycolic acid)
PC	Phosphatidylcholine
CER	Ceramide
CER III	Ceramide III
CER IIIB	Ceramide IIIB
CER VI	Ceramide VI
ROS	Reactive oxygen species
UVA	Ultraviolet A radiation
TNF-α	Tumor necrosis factor alpha
IL-1β	Interleukin-1 beta
IL-6	Interleukin-6
NLRP3	NOD-like receptor family pyrin domain containing 3
VEGF	Vascular endothelial growth factor
TGF-β1	Transforming growth factor beta 1
FT-IR	Fourier transform infrared spectroscopy
DSC	Differential scanning calorimetry
SEM	Scanning electron microscopy
PDI	Polydispersity index
